# Mixed Neurodevelopmental and Neurodegenerative Pathology in *Nhe6*-Null Mouse Model of Christianson Syndrome

**DOI:** 10.1523/ENEURO.0388-17.2017

**Published:** 2018-01-17

**Authors:** Meiyu Xu, Qing Ouyang, Jingyi Gong, Matthew F. Pescosolido, Brandon S. Pruett, Sasmita Mishra, Michael Schmidt, Richard N. Jones, Ece D. Gamsiz Uzun, Sofia B. Lizarraga, Eric M. Morrow

**Affiliations:** 1 Department of Molecular Biology, Cell Biology and Biochemistry, Brown University, Providence, RI 02912; 2 Brown Institute for Brain Science, Brown University, Providence, RI 02912; 3 Developmental Disorders Genetics Research Program, Emma Pendleton Bradley Hospital, East Providence, RI 02915; 4 Department of Psychiatry and Human Behavior, Warren Alpert Medical School of Brown University, Providence, RI 02912; 5 Department of Neurology, Warren Alpert Medical School of Brown University, Providence, RI 02912; 6 Department of Pathology and Laboratory Medicine, Warren Alpert Medical School of Brown University, Providence, RI 02912; 7 Center for Computational Molecular Biology, Brown University, Providence, RI 02912; 8 Department of Biological Sciences, University of South Carolina, Columbia, SC 29208; 9 Center for Childhood Neurotherapeutics, University of South Carolina, Columbia, SC 29208; 10 Hassenfeld Child Health Innovation Institute, Brown University, Providence, RI 02912

**Keywords:** Christianson syndrome, microglia, neurodegeneration, neurodevelopment, NHE6, SLC9A6

## Abstract

Christianson syndrome (CS) is an X-linked disorder resulting from loss-of-function mutations in *SLC9A6*, which encodes the endosomal Na^+^/H^+^ exchanger 6 (NHE6). Symptoms include early developmental delay, seizures, intellectual disability, nonverbal status, autistic features, postnatal microcephaly, and progressive ataxia. Neuronal development is impaired in CS, involving defects in neuronal arborization and synaptogenesis, likely underlying diminished brain growth postnatally. In addition to neurodevelopmental defects, some reports have supported neurodegenerative pathology in CS with age. The objective of this study was to determine the nature of progressive changes in the postnatal brain in *Nhe6*-null mice. We examined the trajectories of brain growth and atrophy in mutant mice from birth until very old age (2 yr). We report trajectories of volume changes in the mutant that likely reflect both brain undergrowth as well as tissue loss. Reductions in volume are first apparent at 2 mo, particularly in the cerebellum, which demonstrates progressive loss of Purkinje cells (PCs). We report PC loss in two distinct *Nhe6*-null mouse models. More widespread reductions in tissue volumes, namely, in the hippocampus, striatum, and cortex, become apparent after 2 mo, largely reflecting delays in growth with more limited tissue losses with aging. Also, we identify pronounced glial responses, particularly in major fiber tracts such as the corpus callosum, where the density of activated astrocytes and microglia are substantially increased. The prominence of the glial response in axonal tracts suggests a primary axonopathy. Importantly, therefore, our data support both neurodevelopmental and degenerative mechanisms in the pathobiology of CS.

## Significance Statement

This study provides evidence for mixed neurodevelopmental and neurodegenerative pathology in mouse models of Christianson syndrome (CS), an X-linked disorder resulting from mutations in the endosomal Na^+^/H^+^ exchanger 6 (NHE6). These data provide important insights for a field where human MRI and postmortem data are limited. Neurodegenerative mechanisms appear prominent in major axon tracts, including a strong microglial response. This study expands on the importance of endosomal processes in neurologic disease. Further, studies of the pathobiology of CS may be relevant to the underlying mechanisms involved in more common brain conditions, both developmental and degenerative. The prominent microglial response raises interest in future preclinical studies in CS guided by efforts to target neuroimmunity in the treatment of neurologic disease.

## Introduction

Monogenic disorders elucidate biological mechanisms with broad relevance to brain function and disease. For neurologic conditions presenting in childhood, studies of disorders such as Rett syndrome and 22q11.2 deletion syndrome have served to elucidate the pathobiology of more common conditions, including autism and schizophrenia (Chahrour and Zoghbi, 2007; Karayiorgou et al., 2010). Increasingly, important questions in the field of neurodevelopmental disorders include the extent to which these rare developmental diseases have progressive brain pathology across the lifespan into adulthood, and how these potentially neurodegenerative mechanisms may inform more common disease. For trisomy 21 (Down syndrome), the existence of both neurodevelopmental and neurodegenerative pathology is well known (Choong et al., 2015). Recent progress in genome-wide sequencing has led to the identification of many new childhood neurogenetic disorders. Our understanding of the consequences of these new mutations in the aging human brain may remain limited until we are able to follow patient cohorts longitudinally. In this way, animal models of disease serve a critical role in elucidating the extent to which these genetic mutations may cause neurodegenerative processes.

The endosomal Na^+^/H^+^ exchanger 6 (NHE6), which is encoded by the X-linked gene *SLC9A6*, is widely expressed. In particular, NHE6 is especially abundant in neurons, where it is implicated in endosomal luminal pH and trafficking, as well as in synapse development and plasticity (Strømme et al., 2011; Deane et al., 2013; Ouyang et al., 2013). The structure of NHE proteins generally involves a 12-membrane-spanning motif harboring the Na^+^/H^+^ exchanger domain, which is highly conserved across NHE family members, and a large, less-conserved carboxyl domain that is thought to mediate protein localization and regulation (Brett et al., 2005; Casey et al., 2010). NHE1–5 are proposed to be localized to the cell membrane, while NHE6–9 are organellar. Endosomal NHEs (NHE6 and NHE9) determine luminal pH by providing a leak pathway for protons, which are pumped in by the vacuolar ATPase (v-ATPase). Loss-of-function mutations in NHE6 cause hyperacidification of the endosomal lumen caused by an imbalance in pump and leak pathways (Ouyang et al., 2013; Ilie et al., 2016).

NHE6 is one of the most recurrently mutated loci in X-linked intellectual disability (Tarpey et al., 2009). Loss-of-function *SLC9A6* mutations cause a neurologic disorder now called Christianson syndrome (CS; formerly known as X-linked Angelman-like syndrome; Gilfillan et al., 2008). CS is characterized by early developmental delay, seizures, ataxia, nonverbal status, and postnatal microcephaly in males (Christianson et al., 1999; Pescosolido et al., 2014). An association with autistic symptoms, including autistic regressions, has also been reported (Christianson et al., 1999; Garbern et al., 2010; Pescosolido et al., 2014). Approximately 90% of boys with CS also meet research criteria for a clinical diagnosis of Angelman syndrome (AS). Parallel to the human developmental phenotypes with NHE6 mutations, NHE6-mutant mice also exhibit developmental defects on a cellular level such as early evidence of reduced neuronal arborization and failures in synapse development *in vitro* and *in vivo* (Ouyang et al., 2013). Signaling mediated by brain-derived neurotrophic factor (BDNF) at the level of signaling endosomes drives dendritic and axonal growth (Greenberg et al., 2009). Thereby, some of the observed developmental phenotypes in both CS and AS—particularly postnatal microcephaly, which is brain undergrowth in the early postnatal period—may be related to decreased endosomal signaling via BDNF (Cao et al., 2013; Ouyang et al., 2013).

In addition to developmental features, CS appears also to be associated with neurodegenerative pathology. One of the most common imaging findings in CS is cerebellar atrophy, which is present in 30%–60% of patients and typically may progress in the second decade of life with worsening ataxia and frequent loss of the ability to walk (Bosemani et al., 2014; Pescosolido et al., 2014). Loss of Purkinje cells (PCs) in the cerebellum with age was previously noted on autopsy by Christianson et al. (1999), and a similar observation was made in a mouse model of CS (Strømme et al., 2011). Later in life, there may be evidence of more widespread neurodegenerative changes. The brains of two middle-aged brothers (i.e., with the same unique in-frame deletion) were examined by MRI and at autopsy. On MRI, moderate generalized brain atrophy and thinning of the corpus callosum (CC) were observed. Post mortem, widespread neuronal loss, gliosis, and neuronal and glial tau pathology were seen (Garbern et al., 2010).

In the current study, we extensively characterize the trajectories of tissue growth and loss in aging *Nhe6*-null mouse brain from birth until very old (2 yr). Given the challenges in following patient cohorts with rare genetic disorders longitudinally and the current scarcity of postmortem material, these data (in this strong animal model) are important for understanding the pathophysiology of CS across the lifespan. Our data are consistent with both brain undergrowth and progressive neurodegenerative processes. These neurodegenerative processes appear first in the cerebellum; however, they present in a more widespread fashion, albeit to a lesser extent across different brain regions, when examined into adulthood. Also, we discover a very strong microglial response, particularly in major axonal tracts, consistent with a primary axonal pathology. The role of microglia and innate immunity has emerged as an important area of neurodegeneration research with therapeutic implications (Heneka et al., 2014; Ransohoff, 2016). Taken together, our observations support both developmental and degenerative processes in CS.

## Materials and Methods

### Animal procedures

Two *Nhe6*-mutant mouse models were used for experiments. In general, matched male wild-type and mutant littermate pairs were used for experiments, as CS is an X-linked condition affecting males. Matched female wild-type and mutant littermate pairs were used for one set of studies (Fig. 8). Numbers of animals used for the various studies are stated in Table 1 and the figure legends. The first mouse model has a *lacZ/Neo* cassette inserted into exon 6. This mouse model has been described previously (Strømme et al., 2011; Ouyang et al., 2013; RRID:MGI:5902071, RRID:IMSR_JAX:005843). A novel *Nhe6*-null mouse model is described herein and was generated by inserting a *lacZ/Neo* cassette to replace exons 2 and 3 of the *Slc9a6* gene (Texas Institute for Genomic Medicine, mouse TG0127). Targeting of the construct was confirmed via PCR genotyping, and loss of NHE6 protein expression was confirmed by Western blot (see below). Both mouse lines were backcrossed for more than seven generations on the C57BL/6J mouse background. All experiments involving live animals were conducted in accordance with the US National Institutes of Health *Guide for the Care and Use of Laboratory Animals* (National Research Council of the National Academies, 2011) under a protocol approved by the Brown University Institutional Animal Care and Use Committee.

**Table 1.** Summary of statistical analyses

			Wild-type (WT)			*Nhe6*-null (MUT)			
Figure, statistical test, and measure	Brain region	Time point, mo	Mean	SEM	*n*	Mean	SEM	*n*	Statistics
Fig. 1									
Two-tailed Student’s **t**test									
Area (cm^2^)									
	Whole brain	0	0.376	0.021	9	0.394	0.011	12	*p* = 0.42
	Whole brain	1	1.015	0.012	11	1.030	0.030	7	*p* = 0.61
	Whole brain	2	1.257	0.033	8	1.112	0.042	6	*p* = 0.018
	Whole brain	6	1.326	0.011	7	1.243	0.011	6	*p* = 0.00025
	Whole brain	23	1.105	0.013	4	1.0068	0.0073	5	*p* = 0.00025
	Cortex	0	0.244	0.013	9	0.2499	0.0083	12	*p* = 0.67
	Cortex	1	0.7527	0.0074	11	0.766	0.020	7	*p* = 0.51
	Cortex	2	0.908	0.027	8	0.808	0.030	6	*p* = 0.029
	Cortex	6	0.949	0.011	7	0.907	0.012	6	*p* = 0.025
	Cortex	23	0.7723	0.0089	4	0.7501	0.0031	5	*p* = 0.035
	Cerebellum	0	0.0316	0.0024	9	0.0323	0.0016	12	*p* = 0.78
	Cerebellum	1	0.2188	0.0050	11	0.2210	0.0088	7	*p* = 0.83
	Cerebellum	2	0.2913	0.0056	8	0.254	0.011	6	*p* = 0.0067
	Cerebellum	6	0.3006	0.0037	7	0.2590	0.0059	6	*p* = 7.5 × 10^–5^
	Cerebellum	23	0.2483	0.0078	4	0.1760	0.0057	5	*p* = 0.00012
	Cerebellum + midbrain	0	0.153	0.014	9	0.1441	0.0029	12	*p* = 0.49
	Cerebellum + midbrain	1	0.2619	0.0062	11	0.264	0.010	7	*p* = 0.85
	Cerebellum + midbrain	2	0.3484	0.0083	8	0.304	0.016	6	*p* = 0.021
	Cerebellum + midbrain	6	0.3776	0.0012	7	0.3365	0.0078	6	*p* = 0.00015
	Cerebellum + midbrain	23	0.3124	0.0064	4	0.2368	0.0068	5	*p* = 0.000096
Fig. 2									
Two-tailed Student’s *t* test									
Thickness (mm)	Cortex	22	1.210	0.029	3	1.090	0.018	3	*p* = 0.025
Area (mm^2^)									
	Striatum	22	4.689	0.030	3	4.062	0.045	3	*p* = 0.00031
	Hippocampus	22	2.455	0.047	3	2.074	0.068	3	*p* = 0.0098
	Cerebellum	22	7.128	0.014	2	4.586	0.089	4	*p* = 4.6 × 10^–5^
Width (mm)									
	Spinal cord (Region 1)	22	1.890	0.073	2	1.491	0.073	2	*p* = 0.060
	Spinal cord (Region 2)	22	1.987	0.080	2	1.845	0.033	2	*p* = 0.24
Fig. 3									
Two-way ANOVA followed by Tukey’s multiple comparison test									
Thickness (mm)									
	Cortex	2	1.154	0.018	3	1.126	0.029	3	*p* = 0.84
	Cortex	22	1.210	0.029	3	1.090	0.018	3	*p* = 0.035
Area (mm^2^)									
	Striatum	2	4.060	0.077	3	3.83	0.12	3	*p* = 0.24
	Striatum	22	4.689	0.030	3	4.062	0.045	3	*p* = 0.0021
	Hippocampus	2	2.199	0.011	3	2.113	0.062	3	*p* = 0.66
	Hippocampus	22	2.455	0.047	3	2.074	0.068	3	*p* = 0.0036
	Cerebellum	2	6.53	0.15	3	5.91	0.16	3	*p* = 0.034
	Cerebellum	22	7.128	0.014	2	4.586	0.089	4	*p* < 0.0001
Slopes generated from linear regression									
Thickness (mm)									
	Cortex	2–22	0.0028	0.0017		–0.0018	0.0017		*p* = 0.10
Area (mm^2^)									
	Striatum	2–22	0.0315	0.0041		0.0116	0.0066		*p* = 0.034
	Hippocampus	2–22	0.0128	0.0024		–0.0020	0.0046		*p* = 0.021
	Cerebellum	2–22	0.0300	0.0097		–0.0661	0.0085		*p* < 0.0001
Fig. 4									
Two-tailed Student’s *t* test									
PC density	Cerebellar vermis: Primary fissure	5	2.94	0.13	3	1.99	0.24	3	*p* = 0.03
Calbindin signal	Cerebellar vermis: Primary fissure	5	32.9	2.8	4	15.5	1.7	4	*p* = 0.002
PC density	Cerebellar vermis: Primary fissure	11–13	3.28	0.28	5	0.77	0.26	4	*p* < 0.001
Calbindin signal	Cerebellar vermis: Primary fissure	11–13	30.7	3.6	6	8.80	0.92	4	*p* = 0.001
PC density	Cerebellar vermis: Primary fissure	5 and 11–13	—	—	—	—	—	—	*p* = 0.006
Calbindin signal	Cerebellar vermis: Primary fissure	5 and 11–13	—	—	—	—	—	—	*p* = 0.01
Fig. 5									
Two-tailed Student’s *t* test									
PC density									
	Cerebellar vermis: Anterior lobe	5	3.27	0.24	3	0.74	0.44	4	*p* = 0.006
	Cerebellar vermis: Flocculonodular lobe	5	3.49	0.36	3	2.42	0.52	3	*p* = 0.17
	Cerebellar vermis: Anterior lobe	11–13	3.22	0.30	4	0.21	0.13	3	*p* < 0.001
	Cerebellar vermis: Flocculonodular lobe	11–13	3.31	0.43	4	2.78	0.51	3	*p* = 0.46
One-way ANOVA followed by Tukey’s multiple comparison test									
PC density	Cerebellar vermis: Primary fissure, Anterior lobe, Flocculonodular lobe	5 and 11–13	—	—	—	—	—	—	11–13 mo MUT *F*_(2,7)_ = 20.11 *p* = 0.001
Fig. 6									
Two-tailed Student’s *t* test									
PC density									
	Periphery	5	4.86	0.45	3	3.95	0.27	3	WT vs. MUT *p* = 0.16
	Periphery	5							Periphery vs. Vermis in WT *p* = 0.02
	Periphery	5							Periphery vs. Vermis in MUT *p* = 0.006
Fig. 7									
Two-tailed Student’s *t* test									
PC density									
	Cerebellar vermis: Primary fissure	6	3.25	0.07	2	1.02	0.38	3	*p* = 0.02
	Cerebellar vermis: Anterior lobe	6	3.80	0.12	2	0.84	0.58	3	*p* = 0.03
	Cerebellar vermis: Flocculonodular lobe	6	3.51	0.43	2	3.24	0.54	3	*p* = 0.75
One-way ANOVA followed by Tukey’s multiple comparison test									
PC density	Cerebellar vermis: Primary fissure, Anterior lobe, Flocculonodular lobe	6	—	—	—	—	—	—	*F*_(2,6)_ = 6.94 *p* = 0.03
Fig. 8									
Two-tailed Student’s *t* test									
PC density	Cerebellar vermis: Primary fissure	5	3.05	0.35	3	0.83	0.45	4	*p* = 0.007
Calbindin signal	Cerebellar vermis: Primary fissure	5	32.1	2.5	3	13.3	1.4	4	*p* = 0.001
Fig. 10									
Two-tailed Student’s *t* test									
Microglia cell count									
	Cortex	22	159	15	3	219	10	4	*p* = 0.019
	Striatum	22	398.7	5.6	3	568	30	4	*p* = 0.0054
	Hippocampus CA	22	74.0	4.4	3	100.0	3.4	4	*p* = 0.0048
	Hippocampus DG	22	56.0	6.7	3	67.3	3.9	4	*p* = 0.18
Fig. 13									
Two-tailed Student’s *t* test									
Size of CD68-positive puncta in Iba1-positive cells (μm^2^)									
	Cortex	22	19.76	0.49	3	28.0	2.0	3	*p* = 0.015
	Striatum	22	20.5	2.0	3	29.9	4.0	3	*p* = 0.11
	Hippocampus DG	22	7.2	2.2	3	10.8	2.5	3	*p* = 0.34

CA, *cornu Ammonis*; CD68, cluster of differentiation 68; DG, dentate gyrus; Iba1, ionized calcium-binding adapter molecule 1; mo, month or months; MUT, *Nhe6*-null; PC, Purkinje cell; SEM, standard error of the mean; WT, wild-type.

### PCR genotyping and Western blotting

To confirm the targeting strategy of the exons 2/3 *Nhe6*-mutant mouse model, PCR genotyping was performed using mouse tail clippings as source samples and the following three primer sequences: (1) exon 3 (5′-ACATACTGCTTCCTCCTATCAT-3′); (2) exon 4 (5′-TTGCTGTTCCAAGAAAGGCATA-3′); and (3) Neo3a target vector (5′-GCAGCGCATCGCCTTCTATC-3′). For Western blot, mouse whole-brain lysates were prepared and boiled at 95°C for 5 min in NuPage 4× LDS sample buffer with NuPage 10× sample reducing agent (Life Technologies). Samples were then subjected to SDS-PAGE in a 4%–12% polyacrylamide gel and transferred to nitrocellulose membranes (Life Technologies) for Western blot analysis according to standard procedures. Briefly, the membrane was blocked for 1 h at room temperature using Odyssey TBS blocking buffer (Licor), incubated overnight at 4°C in primary antibody diluted in TBS blocking buffer, rinsed with TBS-Tween, incubated for 1 h at room temperature in secondary antibody diluted in TBS blocking buffer, rinsed with TBS-Tween, and exposed to detection reagent and X-ray film. Rabbit anti-NHE6 (048; 1:1000 working dilution; Ouyang et al., 2013) and mouse anti-α-tubulin (1:4000 working dilution, Sigma-Aldrich T6074 RRID:AB_477582) primary antibodies and HRP-conjugated mouse anti-rabbit or goat anti-mouse (1:20,000 working dilution; Jackson ImmunoResearch) secondary antibodies were used. SuperSignal West Pico Chemiluminescent Substrate (Thermo Fisher Scientific) was used for detection, and the membrane was exposed to CL-XPosure film (Thermo Fisher Scientific). A Western blot was first performed to detect NHE6, after which the membrane was stripped and reprobed for α-tubulin as a loading control. Stripping was performed for 20 min in NewBlot Nitro Stripping Buffer 5× (Licor) diluted in water to the working concentration, followed by 3 × 5-min washes with 1× PBS.

### Tissue preparation

Mice were anesthetized with Beuthanasia-D and transcardially perfused with 4% paraformaldehyde (PFA). Brains were removed, stored in phosphate-buffered 4% PFA overnight at 4°C, and embedded in 3% low-melting-point agarose.

### Brain size measurement

Matched littermate pairs collected at ages of postnatal day 0 (P0) and 1, 2, 6, and 23–26 mo (23 mo) were transcardially perfused with 4% PFA and imaged using a Leica MZ16F microscope. Gross brain area measures were conducted as described (Ouyang et al., 2016). In brief, ImageJ software (NIH) was used to measure the area of the cortical hemispheres, midbrain, and cerebellum, all while blind to genotype.

### Histology and immunohistochemistry

For Nissl staining, brain sections were 30 μm in thickness, and every 6th section was taken for staining. Five to eight sections were collected for each brain region, and brain regions were confirmed under the microscope by comparing sections to images on the Allen Mouse Brain Atlas site (Allen Institute for Brain Science, 2004). To perform Nissl staining, sections were mounted onto positively charged slides and air-dry fixed. Slides were placed in 0.1% cresyl violet solution for 10 min at 37°C and dehydrated in 95% alcohol and 100% alcohol sequentially. For cerebellar-specific experiments, 50-μm sagittal sections were collected using a Leica Vibratome 100S. For gliosis-specific experiments, free-floating brain sections of 30 μm in thickness were used, with coronal sections used for forebrain analyses and sagittal sections used for hindbrain analyses. To perform immunohistochemistry, sections were first washed 3 times in 1× PBS, permeabilized with 0.1% Triton X-100 for 10 min, blocked with 10% normal goat serum, 1% bovine serum albumin, and 0.1% Triton X-100 in 1× PBS for 2 h at room temperature, and stored in 0.01% sodium azide at 4°C. Sections were subsequently incubated overnight at 4°C with primary antibodies allowing for detection of one or more of the following: calbindin (1:1000 working dilution, mouse, Swant 300 RRID:AB_10000347), neuronal nuclei (NeuN, 1:500 working dilution, mouse, Millipore MAB377 RRID:AB_2298772), ionized calcium-binding adapter molecule 1 (Iba1, 1:500 working dilution, rabbit, Wako 019-19471 RRID:AB_2665520), glial fibrillary acidic protein (GFAP, 1:500 working dilution, chicken, Millipore AB5541 RRID:AB_177521), and/or cluster of differentiation 68 (CD68, 1:100 working dilution, rat, Bio-rad/AbD Serotec MCA1957 RRID:AB_322219). Sections were washed extensively with 1× PBS, after which they were incubated with species-appropriate Alexa Fluor–conjugated secondary antibodies (1:500 or 1:1000 working dilution, Thermo Fisher Scientific) and DAPI (1:1000 working dilution, Thermo Fisher Scientific), if used, for 2 h at room temperature. After washing in 1× PBS, sections were then mounted onto slides using Fluoromount-G (SouthernBiotech).

### Imaging and quantification

Nissl staining was visualized by Zeiss Axiovert 200M bright-field microscopy using a 5× objective. The images were stitched into a whole image and then measured using ImageJ software (NIH) for quantification blind to genotype. Fluorescence microscopy was performed using a Zeiss LSM 710 or Zeiss LSM 800 confocal microscope and a 20× objective. Cerebellar images were acquired as *z*-stacks comprising 20 1-μm slices. PC body density was calculated as the number of PC bodies divided by the length of the PC layer measured. Overall calbindin signal was calculated by measuring the area with positive calbindin signal. The threshold for positive signal was adjusted against the dark background. Images to detect gliosis after immunohistochemical staining were acquired as tile scans so as to have complete images of different brain regions. All images were analyzed using ImageJ software (NIH).

### Stereological section and cell analysis

For Nissl staining of each mouse brain, images were acquired and analyzed blind to genotype from sections of different brain regions as follows: cortex and striatum, 8 images from 4 sequential coronal sections of both left and right hemispheres at coordination 0.26–0.78 mm anterior to Bregma; hippocampus, 6 images from 3 sequential coronal sections of both left and right hemispheres at coordination 1.94–2.18 mm posterior to Bregma; cerebellum, 1 image from 1 midsagittal section; and spinal cord, 1 image from 1 midsagittal section. See Fig. 2 for a depiction of areas of measurement.

For Figs. 4–8, the Sagittal Atlas from the Allen Mouse Brain Atlas was used to determine sagittal sections of the cerebellum to analyze (Allen Institute for Brain Science, 2004). Sections selected for midsagittal analyses most closely resembled Positions 195 and 201 from the Sagittal Atlas, while analyses of cerebellar periphery used sections most similar to Position 74 (http://mouse.brain-map.org/experiment/thumbnails/100042147?image_type=atlas).

For microglia cell counting, images of different brain regions stained for Iba1 using immunohistochemistry were acquired and analyzed blind to genotype. For each brain region, the following number of 228-μm^2^ regions were analyzed for each genotype: cortex, 4 sections with 4 images in each section (16 total); striatum, 4 sections with 9 images in each section (36 total); hippocampus dentate gyrus (DG), 2 sections with 2 images in each section (4 total); and hippocampus *cornu Ammonis* (CA), 2 sections with 3 images in each section (6 total).

### Statistical analysis and modeling

See Table 1 for a summary of statistical analyses relating to this study. Actual *p* values are stated in the text, figure legends, and Table 1; however, for figures, asterisks reflect *p* values as follows: *p ≤ 0.05, **p ≤ 0.01, ***p ≤ 0.001, and ****p ≤ 0.0001. Two-tailed Student’s *t* tests were performed for all group comparisons. For measures of longitudinal changes in brain measures (Fig. 3), two-way ANOVA followed by Tukey’s multiple comparison tests were performed using GraphPad Prism 7 to compare the means for each genotype at each time point to one another. Linear regression analyses were also run on these data to determine significant differences in the slopes of trajectory lines. For cerebellar studies, one-way ANOVA followed by Tukey’s multiple comparison tests were conducted to determine if any mouse genotype had significantly different PC density across cerebellar regions. The between-subjects factor was cerebellar region (e.g., primary fissure, anterior lobe, and flocculonodular lobe). Tukey’s *post hoc* tests were performed with *p* ≤ 0.05 considered to be significant (Figs. 5 and 7). Data are presented as mean ± SEM.

The following mathematical model was used to describe volume change in cortex and cerebellum: dA(t)/dt=GA(t)−DA(t) (adapted from de Pillis et al., 2005). Terms in the equation are defined as follows: *A*(*t*) = area at any time point (cm^2^); $\frac{dA\left(t\right)}{dt}$ = rate of change in area (cm^2^/mo); *G* = growth coefficient (mo^−1^); and *D* = degeneration coefficient (mo^−1^). The following assumptions were made to calculate the coefficients *G* and *D* for stages of 0–1, 1–2, 2–6, and 6–23 mo using experimental data: (1) In wild-type, degeneration rate was assumed to be negligible at 0–6 mo: *D* = 0 at 0 < *t* < 6 mo; and (2) in wild-type, growth rate was assumed to be negligible at 6–23 mo: *G* = 0 at 6 < *t* < 23 mo. The “undergrowth-only” model attempts to explain the differences of the mutant brain sizes based entirely on changes in growth by solving for the required *G* coefficient while maintaining the *D* coefficient at control levels. The “degeneration-only” model attempts to explain the differences of the mutant brain sizes based entirely on accelerated degeneration (without slowed growth) by solving for the required *D* coefficient while maintaining the *G* coefficient at control levels.

## Results

### *Undergrowth and tissue volume loss in* Nhe6-*null brain across postnatal life*

**Figure 1.**
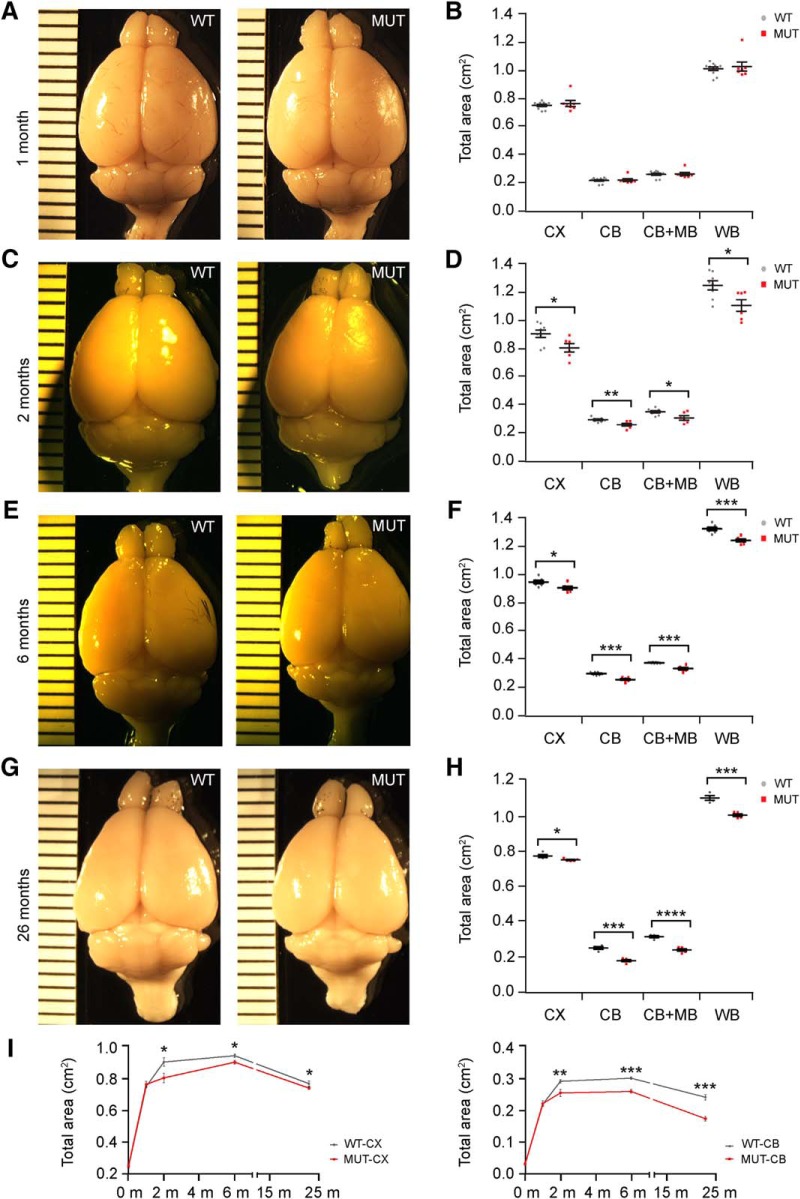
Brain region sizes in wild-type and *Nhe6*-null mice across the postnatal lifespan. ***A***, ***C***, ***E***, ***G***, Representative images of brains from wild-type (WT) and *Nhe6*-null (MUT) male littermates at 1, 2, 6, and 26 mo, respectively. Ruler markers represent millimeters. ***B***, ***D***, ***F***, ***H***, Graphs depicting quantitative analysis of total areas for whole brain and different brain regions at time points corresponding to respective images to the left. There are no significant differences in brain size between WT and *Nhe6*-null mice at 1 mo. For total areas at 2, 6, and 23–26 mo, male *Nhe6*-null mice exhibit significantly decreased whole-brain area (*, *p* = 0.018; ***, *p* = 0.00025; and ***, *p* = 0.00025, respectively), as well as cortical area (*, *p* = 0.029; *, *p* = 0.025; and *, *p* = 0.035, respectively), cerebellar area (**, *p* = 0.0067; ***, *p* = 7.5 × 10^–5^; and ***, *p* = 0.00012, respectively), and cerebellar + midbrain area (*, *p* = 0.021; ***, *p* = 0.00015; and ****, *p* = 0.000096, respectively), compared with male WT mice. CX, cortex; CB, cerebellum; MB, midbrain; WB, whole brain. ***I***, Graphs depicting longitudinal representation of total areas for cortex (left) and cerebellum (right) in WT versus MUT mice of the indicated ages in months (m). Time points of analysis were P0 and 1, 2, 6, and 23–26 mo, which is plotted as 23 mo. WT *n* = 11, MUT *n* = 7 (***B***); WT *n* = 8, MUT *n* = 6 (***D***); WT *n* = 7, MUT *n* = 6 (***F***); WT *n* = 4, MUT *n* = 5 (***H***). Data are presented as mean ± SEM. Statistical analyses were conducted using two-tailed Student’s *t* tests.

To understand the trajectories of brain volume changes across the lifespan in *Nhe6*-null male mice, we collected brains from male mice at P0; 1, 2, and 6 mo; and ∼2 yr of age (23–26 mo). Direct measurement of gross brain size reveals that the brain sizes are similar in *Nhe6*-null and wild-type littermates at the initial stages of postnatal brain growth, namely, at P0 (Table 1) and 1 mo (Fig. 1*A*, *B*). By gross measure, the earliest apparent differences emerge by 2 mo, when both the cerebrum (CX, cortical region) and the cerebellum (CB) are smaller in mutant animals compared with wild-type littermates (Fig. 1*C*, *D*). Although there is continued growth of the cerebrum in *Nhe6*-null animals, a reduction in the rate of increase exists at 1–2 mo as well as reduced size in mutants overall. The cerebrum of mutant animals remains significantly smaller at 6 mo, the peak in size in both mutant and control (Fig. 1*E*, *F*). Between 6 mo and 2 yr of age (Fig. 1*G*, *H*), there are reductions in volumes in both the mutant and control. In the cerebrum, slopes of loss are similar in mutant and control such that the mutant remains significantly smaller into old age (Fig. 1*I*). In addition, the brain size in *Nhe6*-null male mice remains significantly smaller in all regions measured at 2 yr (Fig. 1*G*, *H*). See Table 1 for a summary of statistical analyses for these and all subsequent experiments.

Trajectories of changes in cerebral and cerebellar gross brain volumes across the lifespan in male *Nhe6*-null mice are shown in Fig. 1*I*. Cerebral brain volume changes in wild-type male mice and *Nhe6*-null male mice from P0 to ∼2 yr, as reflected by our analyses, are summarized as follows. In wild-type male mice: P0 to 1 mo, statistically significant increase (*p* = 3.7 × 10^–18^); 1–2 mo, statistically significant increase (*p* = 7.0 × 10^–6^); 2–6 mo, relative plateau with no statistically significant change (*p* = 0.21); and 6–23 mo, statistically significant decrease (*p* = 1.7 × 10^–6^). In *Nhe6*-null male mice: P0 to 1 mo, statistically significant increase (*p* = 6.4 × 10^–16^); 1–2 mo, relative plateau with no statistically significant change (*p* = 0.28); 2–6 mo, slow growth yet with a statistically significant increase indicating delays in growth with some catching up toward wild-type animal size (*p* = 0.012); and 6–23 mo, statistically significant decrease (*p* = 8.9 × 10^–7^). Notably, these trajectories reveal important information suggesting that the reduction in cerebral volume in the mutant may reflect a large component of undergrowth.

The trajectories of cerebellar volume suggest a stronger degenerative component. Here, wild-type and mutant animals display similar brain growth trajectories from P0 to 1 mo; however, mutant animals show a decreased rate of increase from 1 to 2 mo compared with wild-type animals, with a subsequent relative plateau from 2 to 6 mo. From 6 to 23 mo, cerebellar volume decreases severely in mutant mice at a greater rate than the control, indicative of a neurodegenerative phenotype in *Nhe6*-null male cerebellum. Cerebellar brain volume changes in wild-type male mice and *Nhe6*-null male mice from P0 to ∼2 yr, as reflected by our analyses, are summarized as follows. In wild-type male mice: P0 to 1 mo, statistically significant increase (*p* = 8.0 × 10^–17^); 1–2 mo, statistically significant increase (*p* = 4.8 × 10^–8^); 2–6 mo, relative plateau with no statistically significant change (*p* = 0.20); and 6–23 mo, statistically significant decrease (*p* = 6.9 × 10^–5^). In *Nhe6*-null male mice: P0 to 1 mo, statistically significant increase (*p* = 5.3 × 10^–15^); 1–2 mo, statistically significant increase (*p* = 0.041); 2–6 mo, relative plateau with no statistically significant change (*p* = 0.70); and 6–23 mo, statistically significant decrease (*p* = 3.8 × 10^–6^).

We conducted mathematical modeling to describe the growth and degeneration rates in wild-type and *Nhe6*-null male cerebrum and cerebellum (Table 2), using an equation adapted from a prior publication (see Materials and Methods and de Pillis et al., 2005). Assuming control growth occurs at 0–6 mo and degeneration at 6–23 mo, we calculated coefficients of growth (*G*) and degeneration (*D*) for the wild-type cerebrum and cerebellum. We then considered the data for an undergrowth-only model wherein differences in size in the mutant are explained entirely by reductions in growth by calculating coefficients *G* after setting the degeneration coefficient *D* to wild-type levels. Also, we considered the data under a degeneration-only model wherein differences in size in the mutant are explained entirely by enhanced degeneration alone (without invoking undergrowth) after setting the growth coefficient *G* to wild-type levels. Neither the cerebrum or the cerebellum data fit the degeneration-only model without invoking two distinct phases of strong degeneration, which seems less plausible than a mixed model involving an early phase of undergrowth superimposed by a later phase of accelerated degeneration (see Discussion). The cerebrum data do fit an undergrowth-only model, with a similar degenerative rate as wild-type animals. The cerebellum strongly supports a mixed scenario of both undergrowth and enhanced neurodegeneration, as the undergrowth-only model fits the data at 0–6 mo, but enhanced neurodegeneration at 6–23 mo is required to fit the data.

**Table 2.** Modeling of cortex and cerebellum growth and degeneration

	Wild-type (WT)			*Nhe6*-null (MUT)				
	Rate (cm^2^/mo)	Coefficients (mo^−1^)		Rate (cm^2^/mo)	Undergrowth-only modelCoefficients (mo^−1^)		Degeneration-only modelCoefficients (mo^−1^)	
Brain region and time point	Mean	*G*	*D*	Mean	*G*	*D*	*G*	*D*
Cortex								
0–1 mo	0.5092	0.6764	0	0.516	0.6737	0	0.6764	0.0027
1–2 mo	0.156	0.1712	0	0.0415	0.0514	0	0.1712	0.1198
2–6 mo	0.0101	0.0106	0	0.0248	0.0274	0	0.0106	–0.0168
6–23 mo	–0.01037	0	0.0134	–0.00922	0	0.0123	0	0.0123
Cerebellum								
0–1 mo	0.1873	0.8558	0	0.1887	0.8537	0	0.8558	0.0021
1–2 mo	0.07243	0.2487	0	0.0332	0.1305	0	0.2487	0.1182
2–6 mo	0.00233	0.0078	0	0.0012	0.0047	0	0.0078	0.0031
6–23 mo	–0.00308	0	0.0124	–0.00488	0	0.0277	0	0.0277

*D*, Degeneration coefficient; *G*, Growth coefficient; mo, month or months; MUT, *Nhe6*-null; WT, wild-type.

**Figure 2.**
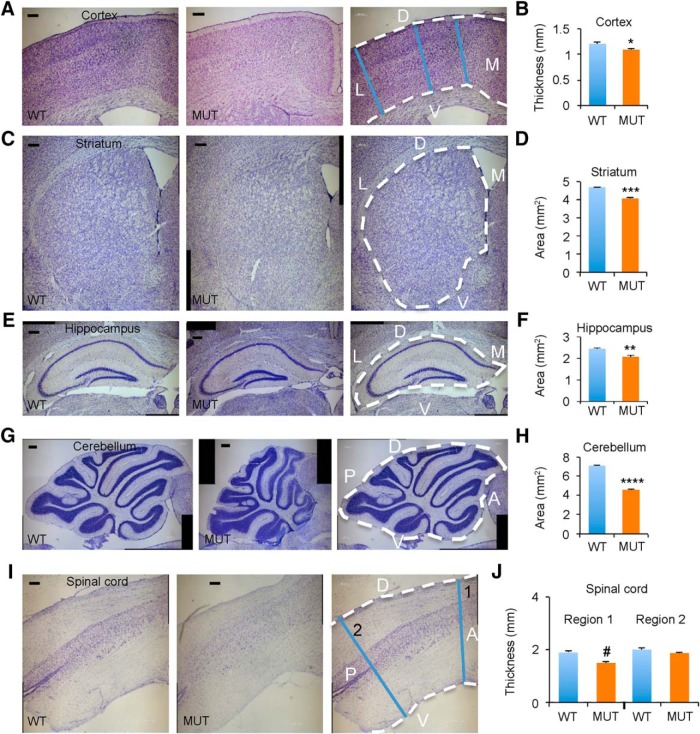
Widespread reduction of neural tissue in aged *Nhe6*-null mice. ***A***, ***C***, ***E***, ***G***, ***I***, Representative images of 30-μm brain sections from 22-mo-old WT and MUT male littermate mice after Nissl staining. Shown are coronal brain sections of cortex (***A***), striatum (***C***), and hippocampus (***E***) and sagittal sections of cerebellum (***G***) and spinal cord (***I***). Rightmost panels depict sections overlaid with anatomic labels for orientation purposes and blue lines indicating regions of measurement for assessment of atrophy. Two different anterior-posterior thicknesses of spinal cord (1 and 2) are shown and were analyzed (***I***). D, dorsal; L, lateral; V, ventral; M, medial; A, anterior; P, posterior. Scale bars, 200 μm. ***B***, ***D***, ***F***, ***H***, ***J***, Graphs depicting quantification of thicknesses or areas of different brain regions in MUT male mice compared with WT male littermates, with each graph corresponding to the brain region reflected in the images to its left. WT *n* = 3, MUT *n* = 3 (***B***, ***D***, and ***F***); WT *n* = 2, MUT *n* = 4 (***H***); WT *n* = 2, MUT *n* = 2 (***J***). Data are presented as mean ± SEM. Statistical analyses were conducted using two-tailed Student’s *t* tests. *, *p* = 0.025 (***B***); ***, *p* = 0.00031 (***D***); **, *p* = 0.0098 (***F***); ****, *p* = 4.6 × 10^–5^ (***H***); #, *p* = 0.060 (***J***, Region 1); *p* = 0.24 (***J***, Region 2).

To identify specific regions of reduced tissue volume with aging, measurements were conducted using Nissl-stained brain sections from *Nhe6*-null male mice and wild-type littermates at 22 mo of age (Fig. 2). Widespread reductions in the thickness or area of different major brain regions are present in *Nhe6*-null male mice relative to wild-type littermates at this time point, particularly in the cerebellum. Quantitative measurements of different brain regions in wild-type versus *Nhe6*-null male mice reveal the following, with statistical analyses performed using two-tailed Student’s *t* tests (Fig. 2 and Table 1): cortex, 10% ± 1.5% decrease in thickness (Fig. 2*A*, *B*, *p* = 0.025); striatum, 13% ± 1% decrease in area (Fig. 2*C*, *D*, *p* = 0.00031); hippocampus, 15% ± 3% decrease in area (Fig. 2*E*, *F*, *p* = 0.0098); cerebellum, 36% ± 1% decrease in area (Fig. 2*G*, *H*, *p* = 4.6 × 10^–5^); and spinal cord, 21% ± 4% decrease in thickness (Fig. 2*I*, *J*, *p* = 0.060). These results strongly support a model wherein there is widely distributed reduction in brain growth and/or loss of brain tissue progressing into adulthood in male *Nhe6*-null brains.

**Figure 3.**
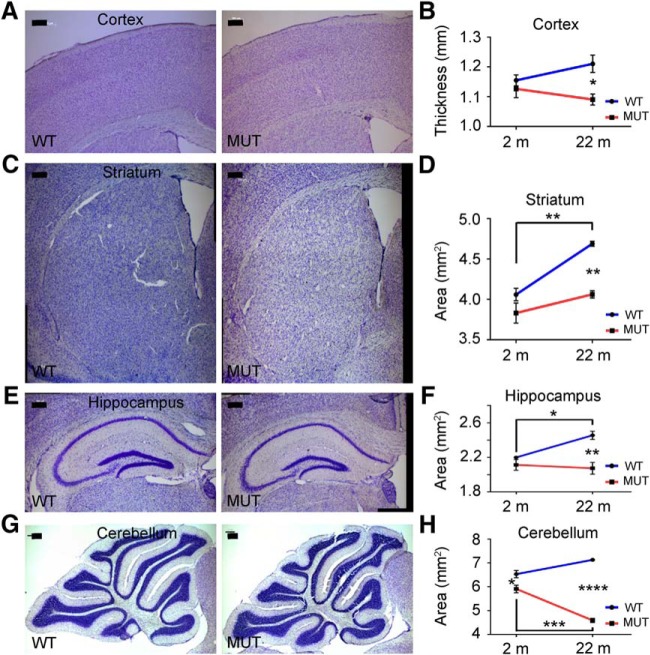
Trajectories of brain tissue changes in *Nhe6*-null mice from 2 mo to 2 yr. ***A***, ***C***, ***E***, ***G***, Representative images of 30-μm brain sections from 2-mo-old WT and MUT male littermate mice after Nissl staining. Coronal sections were used for cortex (***A***), striatum (***C***), and hippocampus (***E***), and sagittal sections were used for cerebellum (***G***). Measurements were performed as in Fig. 2. Scale bars, 200 μm. ***B***, ***D***, ***F***, ***H***, Graphs depicting quantification of thicknesses or areas of different brain regions in MUT male mice compared with WT male littermates at time points of 2 months (2 m) and 22 months (22 m; from Fig. 2). Each graph corresponds to the brain region reflected in the images to its left. WT *n* = 3, MUT *n* = 3 for each age group (***B***, ***D***, and ***F***); WT *n* = 3, MUT *n* = 3 for 2 m and WT *n* = 2, MUT *n* = 4 for 22 m (***H***). Data are presented as mean ± SEM. Statistical analyses were conducted using two-way ANOVA followed by Tukey’s multiple comparison tests to compare the means for each genotype at each time point to one another. See Results for details. *, *p* = 0.035 (***B***); **, *p* = 0.0020 (WT 2 m to 22 m; ***D***); **, *p* = 0.0021 (22 m; ***D***); *, *p* = 0.033 (WT 2 m to 22 m; ***F***); **, *p* = 0.0036 (22 m; ***F***); *, *p* = 0.034 (2 m; ***H***); ***, *p* = 0.0002 (MUT 2 m to 22 m; ***H***); ****, *p* < 0.0001 (22 m; ***H***).

To better understand the onset and trajectories of changes in brain size described above relating to 22-mo-old mice, we examined Nissl-stained brain sections from 2-mo-old *Nhe6*-null male mice and wild-type littermates (Fig. 3). Data from 2-mo-old mice were compared with those from 22-mo-old mice using two-way ANOVA followed by Tukey’s multiple comparison tests (Fig. 3*B*, *D*, *F*, *H* and Table 1). Considering genotype and age, a significant overall effect of mutant genotype is present with respect to all four of the major brain regions measured, including cortical thickness [Fig. 3*B*, accounting for 44.08% of total variance, *F*_(1,8)_ = 9.16, *p* = 0.016]; striatal area [Fig. 3*D*, accounting for 40.15% of total variance, *F*_(1,8)_ = 30.15, *p* = 0.0006]; hippocampal area [Fig. 3*F*, accounting for 49.85% of total variance, *F*_(1,8)_ = 20.4, *p* = 0.0020]; and cerebellar area [Fig. 3*H*, accounting for 61.8% of total variance, *F*_(1,8)_ = 147.1, *p* < 0.0001], with the latter region revealing the most pronounced effect. The analyses also reveal a significant effect of age on striatal area [Fig. 3*D*, accounting for 40.54% of total variance, *F*_(1,8)_ = 30.44, *p* = 0.0006] and cerebellar area [Fig. 3*H*, accounting for 3.23% of total variance, *F*_(1,8)_ = 7.675, *p* = 0.024], as well as a significant interaction between genotype and age for striatal area [Fig. 3*D*, accounting for 8.65% of total variance, *F*_(1,8)_ = 6.492, *p* = 0.034], hippocampal area [Fig. 3*F*, accounting for 19.91% of total variance, *F*_(1,8)_ = 8.15 *p* = 0.021], and cerebellar area [Fig. 3*H*, accounting for 22.8% of total variance, *F*_(1,8)_ = 54.27, *p* < 0.0001].

*Post hoc* analyses focusing on the earlier time point of 2 mo demonstrate a small but significant decrease in cerebellar tissue area in mutant animals relative to wild-type (Fig. 3*G*, *H*, *p* = 0.034), but no statistically significant reduction of tissue thickness in cortex (Fig. 3*A*, *B*, *p* = 0.84), striatum (Fig. 3*C*, *D*, *p* = 0.24), or hippocampus (Fig. 3*E*, *F*, *p* = 0.66). Similar to the findings presented in Fig. 2, *post hoc* analyses focusing on the 22-mo time point again demonstrate significant differences between *Nhe6*-null male mice and wild-type littermates in cortical thickness (Fig. 3*B*, *p* = 0.035), striatal area (Fig. 3*D*, *p* = 0.0021), hippocampal area (Fig. 3*F*, *p* = 0.0036), and cerebellar area (Fig. 3*H*, *p* < 0.0001), with thickness or area in all four regions being significantly less in *Nhe6*-null male mice. Focusing within the genotypes, *post hoc* analyses also reveal a significant age-related increase in areas of the striatum (Fig. 3*D*, *p* = 0.0020) and hippocampus (Fig. 3*F*, *p* = 0.033) in wild-type mice, but not in *Nhe6*-null male mice, reflecting undergrowth in these regions in the mutant. Mutant animals display a relative plateau, i.e., no significant change, in area at 2–22 mo in striatum (Fig. 3*D*, *p* = 0.23) and hippocampus (Fig. 3*F*, *p* = 0.95). There was no significant interval change in thickness of cortex in either the wild-type or mutant, although there was a small upward trend in wild-type mice and downward trend in mutant mice (Fig. 3*B*). Furthermore, *Nhe6*-null male mice display a significant reduction in cerebellar tissue area at 2–22 mo (Fig. 3*H*, *p* = 0.0002), a reduction not observed in wild-type male littermates during the same interval.

To further assess trajectories of brain changes between the genotypes, we also subjected the slope (rate of change from 2 to 22 mo) to linear regression analyses (Table 1). Based on these analyses, the rates of change in brain region thickness or area (i.e., slopes) from 2 to 22 mo are significantly lower in *Nhe6*-null male mice compared with wild-type male littermates for striatal area (*p* = 0.034), hippocampal area (*p* = 0.021), and in particular, cerebellar area (*p* < 0.0001), but not for cortical thickness (*p* = 0.10). These data are quite informative, as we discern clearly distinct trajectories of change in different brain regions, with the most marked downward slope in the cerebellum. In the cortex, striatum, and hippocampus, trajectories of changes (as revealed by both gross and histologic measures) appear to reflect a prominent component of undergrowth.

### *Temporal trajectories and anatomic patterns of Purkinje cell loss in two distinct* Nhe6-*null mouse lines*

**Figure 4.**
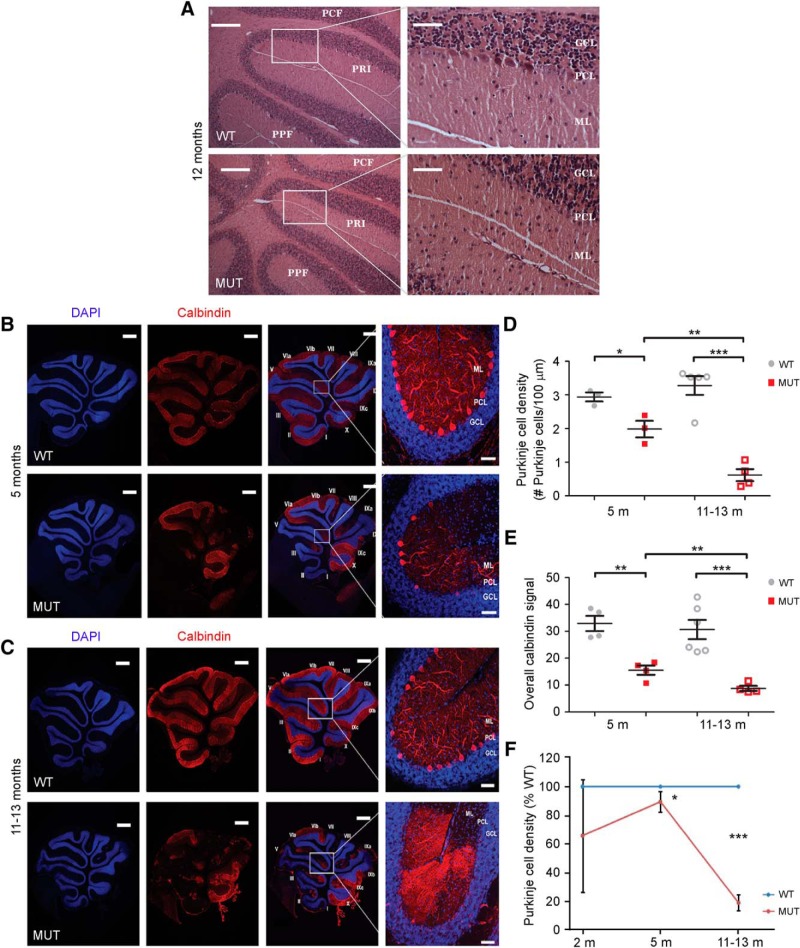
Temporal progression of PC loss in the primary fissure of the vermis in *Nhe6*-null cerebellum. ***A***, Representative images of hematoxylin and eosin-stained midsagittal sections of the vermal primary fissure from 12-mo-old WT and MUT male mice. PCF, preculminate fissure; PRI, primary fissure; PPF, prepyramidal fissure; ML, molecular layer; PCL, Purkinje cell layer; GCL, granule cell layer. Scale bars, 200 μm (left panels) and 50 μm (right panels). ***B***, ***C***, Representative images of midsagittal sections of the vermal primary fissure immunostained with the PC marker calbindin (red) in WT and MUT male mice at ages 5 mo (***B***) and 11–13 mo (***C***). Nuclei were stained with DAPI (blue). Vermis folia are labeled with Roman numerals according to the Allen Mouse Brain Atlas (Lein et al., 2007). Scale bars, 50 μm. ***D***, ***E***, Graphs depicting quantification of PC density (***D***) and overall calbindin signal (***E***) in WT and MUT male mice 5 months of age (5 m) and 11–13 months of age (11–13 m). *Nhe6*-null male mice (MUT) display significantly decreased PC density at both 5 m (*, *p* = 0.03) and 11–13 m (***, *p* < 0.001), as well as significantly decreased overall calbindin signal at both ages (**, *p* = 0.002, 5 m; ***, *p* = 0.001, 11–13 m). Furthermore, *Nhe6*-null mice exhibit a progressive decrease in PC density (**, *p* = 0.006, ***D***) and overall calbindin signal (**, *p* = 0.01, ***E***) over the 5- to 11–13-mo time period, a decrease that was not observed in WT male mice. ***F***, Graph depicting the trajectory of PC density of MUT male mice in comparison to age-matched WT male mice based on data collected from mice 2, 5, or 11–13 mo. WT *n* = 3, MUT *n* = 3 for 5 m and WT *n* = 5, MUT *n* = 4 for 11–13 m (***D*** and ***F***); WT *n* = 4, MUT *n* = 4 for 5 m and WT *n* = 6, MUT *n* = 4 for 11–13 m (***E***); WT *n* = 2, MUT *n* = 2 for 2 m (***F***). Data are presented as mean ± SEM. Statistical analyses were conducted using two-tailed Student’s *t* tests.

Cerebellar pathology represents one of the most robust findings in CS (Christianson et al., 1999; Strømme et al., 2011; Pescosolido et al., 2014) and may constitute the earliest neurodegenerative finding. Severe PC loss, indicative of neurodegeneration, has been noted in postmortem examinations of male patients with *NHE6* mutations and in an *Nhe6*-mutant mouse (Christianson et al., 1999; Garbern et al., 2010). Here, we expand on these prior findings of PC loss and replicate this finding in a new and distinct *Nhe6*-mutant mouse (described below). First, at 12 mo of age using hematoxylin and eosin staining of midsagittal cerebellar sections, *Nhe6*-null male mice exhibit a striking reduction of PCs in the primary fissure of the vermis compared to wild-type male mice (Fig. 4*A*). We next quantified the temporal course of PC loss using anti-calbindin immunohistochemistry, a specific PC marker, as applied to midsagittal sections of the primary fissure of the vermis. *Nhe6*-null male mice exhibit decreased PC density and overall calbindin signal in the vermal primary fissure, a phenotype evident at 5 mo and progressively worse at 11–13 mo (Fig. 4*B–F*). At 5 mo, PC density and overall calbindin signal is significantly decreased in *Nhe6*-null male mice compared with wild-type male littermates (Fig. 4*D*, *E*, *p* = 0.03 and *p* = 0.002, respectively). *Nhe6*-null male mice 11–13 mo of age also demonstrate significant PC loss relative to wild-type male littermates, as reflected by quantification of PC density and overall calbindin signal (Fig. 4*D*, *E*, *p* < 0.001 and *p* = 0.001, respectively). PC loss may begin before 5 mo; however, results from 2-mo-old mice were highly variable and not statistically significant (Fig. 4*F*). These results suggest that *Nhe6*-null male mice display progressive PC loss largely starting after 2 mo of age and worsening from 5 to 11–13 mo.

**Figure 5.**
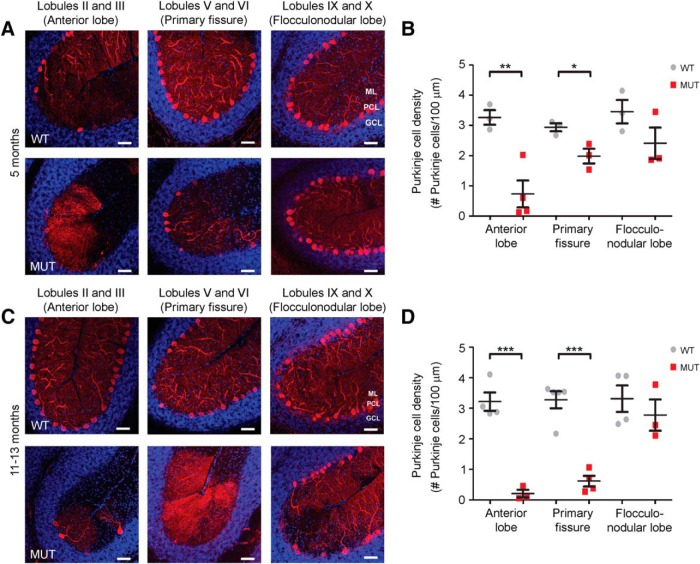
Anatomic patterns of PC loss in *Nhe6*-null cerebellum. ***A***, ***C***, Representative images of midsagittal sections of the anterior lobe (lobules II and III), vermal primary fissure (lobules V and VI), and flocculonodular lobe (lobules IX and X) immunostained with the PC marker calbindin (red) in WT and MUT male mice at ages 5 mo (***A***) and 11–13 mo (***C***). Nuclei were stained with DAPI (blue). ML, molecular layer; PCL, Purkinje cell layer; GCL, granule cell layer. Scale bars, 50 μm. ***B***, ***D***, Graphs depicting quantification of PC density across cerebellar regions in WT and MUT male mice 5 mo (***B***) and 11–13 mo (***D***). At 5 mo, PC density is significantly decreased in the anterior lobe (**, *p* = 0.006) and primary fissure (*, *p* = 0.03, previously shown in Fig. 4*B*, *D*), but not the flocculonodular lobe (*p* = 0.17), in MUT male mice compared with WT male mice (***B***). Similarly, at 11–13 mo, MUT male mice show significantly decreased PC density in the anterior lobe (***, *p* < 0.001) and primary fissure (***, *p* < 0.001, previously shown in Fig. 4*C*, *D*), but not the flocculonodular lobe (*p* = 0.46), relative to WT male mice (***D***). Also, there is a statistically significant difference between cerebellar regions in *Nhe6*-null mice 11–13 mo of age [*F*_(2,7)_ = 20.11, *p* = 0.001], but not in *Nhe6*-null mice 5 mo of age. WT *n* = 3, MUT *n* = 3 for vermal primary fissure and flocculonodular lobe (***B***); WT *n* = 3, MUT *n* = 4 for anterior lobe (***B***); WT *n* = 4, MUT *n* = 3 for anterior lobe and flocculonodular lobe (***D***); WT *n* = 5, MUT *n* = 4 for vermal primary fissure (***D***). Data are presented as mean ± SEM. Statistical analyses were conducted using two-tailed Student’s *t* tests for group comparisons and one-way ANOVA followed by Tukey’s multiple comparison tests for cerebellar region comparisons. Note that representative vermal primary fissure images from mice at ages 5 mo (***A***) and 11–13 mo (***C***) are the same as those for the respective time points in Fig. 4*B*, *C*.

**Figure 6.**
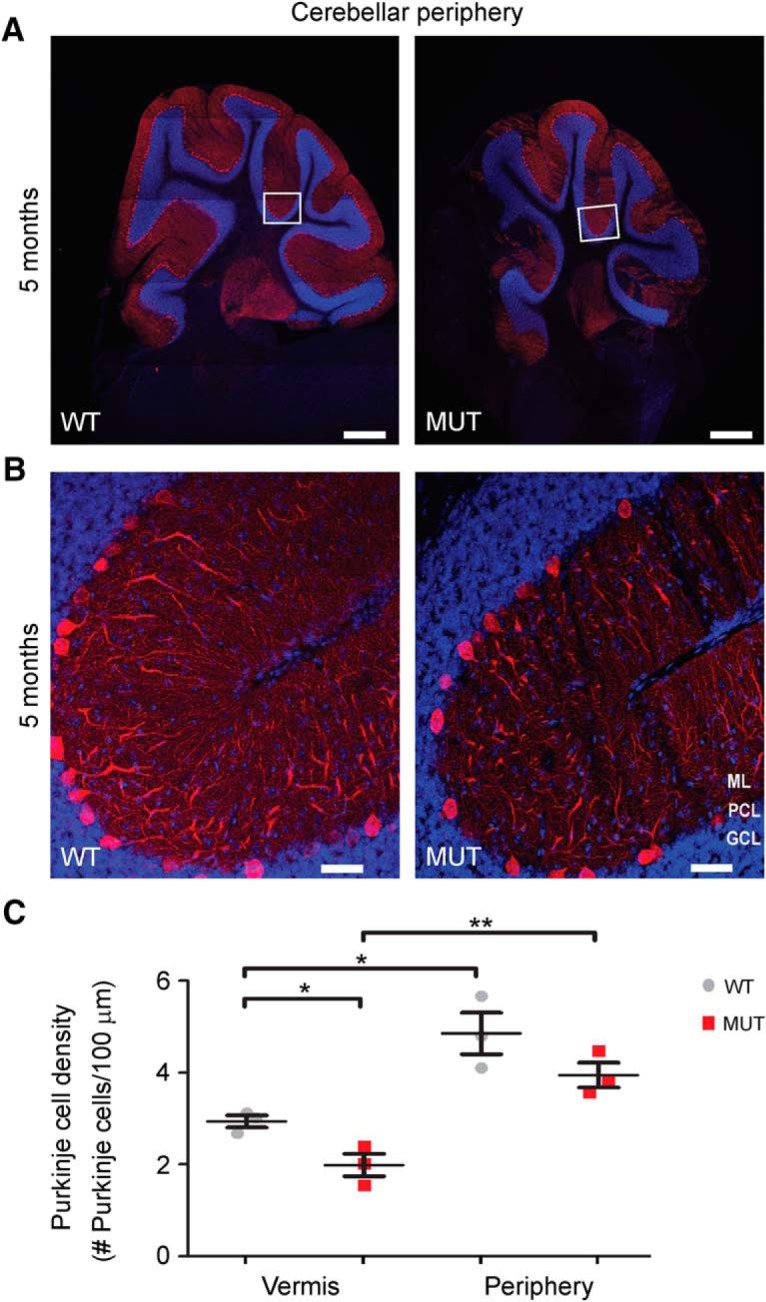
PC loss in the cerebellar vermis as opposed to lateral regions. ***A***, ***B***, Representative images of sagittal sections of whole cerebellar periphery immunostained with the PC marker calbindin (red) in WT and MUT male mice at 5 mo of age. Images in ***B*** reflect higher magnification of the respective boxed regions in ***A***. Nuclei were stained with DAPI (blue). ML, molecular layer; PCL, Purkinje cell layer; GCL, granule cell layer. Scale bars, 500 μm (***A***) and 50 μm (***B***). ***C***, Graph depicting quantitative analysis of PC density in the cerebellar vermis (from Fig. 4*D*) versus the cerebellar periphery in WT and MUT male mice of 5 mo of age. Differences in PC density in peripheral regions of the cerebellum are not detected in WT versus MUT male mice at this time point (*p* = 0.16). However, the peripheral cerebellum shows a significantly greater PC density compared with the cerebellar vermis at 5 mo in both WT mice (*, *p* = 0.02) and MUT mice (**, *p* = 0.006). As shown in Figs. 4 and 5, *Nhe6*-null male mice (MUT) at 5 mo of age display significantly decreased PC density in the primary fissure of the vermis (*, *p* = 0.03). WT *n* = 3, MUT *n* = 3 for each cerebellar region analyzed. Data are presented as mean ± SEM. Statistical analyses were conducted using two-tailed Student’s *t* tests.

We next asked whether PC loss might also occur in other cerebellar regions beyond the primary fissure of the vermis, such as in the anterior lobe and flocculonodular lobe. Using midsagittal sections along the vermis for examination, *Nhe6*-null male mice 5 mo of age exhibit significantly decreased PC density in the anterior lobe (*p* = 0.006), but not in the flocculonodular lobe (*p* = 0.17), compared with wild-type male littermates (Fig. 5*A*, *B*). A similar phenotype is present in *Nhe6*-null male mice 11–13 mo of age, namely, a statistically significant decrease in PC density in the anterior lobe (*p* < 0.001), but not in the flocculonodular lobe (*p* = 0.46), relative to wild-type male littermates (Fig. 5*C*, *D*). One-way ANOVA followed by Tukey’s multiple comparison tests reveal a statistically significant difference in PC density across cerebellar regions in *Nhe6*-null mice 11–13 mo of age, but not in mice 5 mo of age; the mean PC density of both the primary fissure and anterior lobe differs significantly from the flocculonodular lobe [*F*_(2,7)_ = 20.11, *p* = 0.001]. In addition to results regarding PC loss in the cerebellar vermis, we also observe a mild loss of PCs in *Nhe6*-null male mice compared with wild-type male littermates in the primary fissure in lateral cerebellum. However, the PC loss in sagittal sections of lateral cerebellar regions is more mild and not statistically significant (Fig. 6 and Table 1).

**Figure 7.**
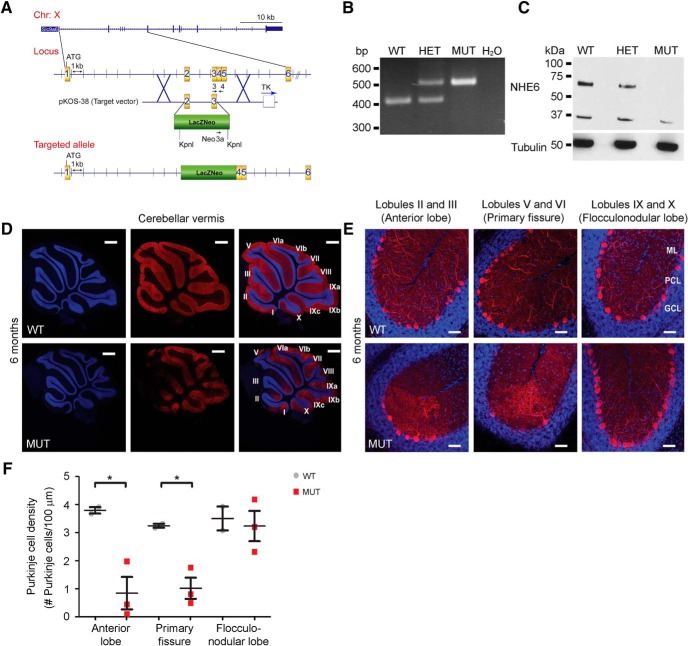
Anatomic patterns of cerebellar PC loss in a new exons 2/3 *Nhe6*-null mouse model. ***A***, Schematic of the targeting approach and vector for generating the exons 2/3 *Nhe6*-null mouse model. ***B***, ***C***, Loss of *Nhe6* mRNA and NHE6 protein expression in the exons 2/3 *Nhe6*-null mouse model was confirmed via PCR, using mouse tail clippings as a sample source (***B***), and via Western blot, using whole-brain lysate as a sample source (***C***), respectively. For Western blotting, the membrane was first probed to detect NHE6, after which the membrane was stripped and reprobed for α-tubulin as a loading control. WT, wild-type; HET, heterozygous; MUT, exons 2/3 *Nhe6*-null mutant. ***D***, ***E***, Representative images of midsagittal sections of whole cerebellar vermis immunostained with the PC marker calbindin (red) in WT and exons 2/3 *Nhe6*-null MUT male mice at 6 mo of age. Lower-magnification images depicting lobules I through X are shown in ***D***, and higher-magnification images depicting specific lobules and regions as indicated are shown in ***E***. Nuclei were stained with DAPI (blue). ML, molecular layer; PCL, Purkinje cell layer; GCL, granule cell layer. Scale bars, 500 μm (***D***) and 50 μm (***E***). ***F***, Graph depicting quantification of PC density across cerebellar regions in WT and MUT male mice at 6 mo of age. Compared with WT males, exons 2/3 MUT male mice exhibit significantly decreased PC density in the anterior lobe (*, *p* = 0.03) and primary fissure (*, *p* = 0.02), yet a statistically significant difference is not observed in the flocculonodular lobe (*p* = 0.75). Furthermore, MUT male mice exhibit a region-specific decrease in PC density in the primary fissure and anterior lobe, but not in the flocculonodular lobe [*F*_(2,6)_ = 6.94, *p* = 0.03]. WT *n* = 2, MUT *n* = 3. Data are presented as mean ± SEM. Statistical analyses were conducted using two-tailed Student’s *t* tests for group comparisons and one-way ANOVA followed by Tukey’s multiple comparison tests for cerebellar region comparisons.

To date, all published studies of *Nhe6*-null mice have examined the same mutant model, which is based on introduction of a *lacZ*/*Neo* cassette into exon 6, thereby producing a stop codon after the proton exchanger domain (Strømme et al., 2011; Ouyang et al., 2013). To further support that findings from the exon 6 *Nhe6*-null mouse model are in fact due to mutations in *Nhe6* and independent of genetic background, we established a novel *Nhe6*-null mouse model with a distinct gene targeting event. Namely, a *lacZ/Neo* cassette was inserted into exons 2/3; this insertion would be predicted to cause an early truncation before the NHE6 exchanger domain and/or nonsense-mediated mRNA decay (Fig. 7*A*). PCR genotyping and Western blot confirm the gene targeting approach and loss of NHE6 protein expression, respectively, in the exons 2/3 *Nhe6*-null mouse model (Fig. 7*B*, *C*).

Importantly, exons 2/3 *Nhe6*-null male mice show an identical phenotype as the exon 6 *Nhe6*-null male mice with respect to the level and distinct pattern in PC loss. Here, in comparison to wild-type male littermates, 6-mo-old exons 2/3 *Nhe6*-null male mice show a significant reduction in PC density in the primary fissure (*p* = 0.02) and anterior lobe (*p* = 0.03), but not in the flocculonodular lobe (*p* = 0.75), in midsagittal sections (Fig. 7*D–F*). Also, one-way ANOVA followed by Tukey’s multiple comparison tests reveal a statistically significant region-specific decrease in cerebellar PC density in exons 2/3 *Nhe6*-null mice of 6 mo of age; the mean PC density of both the primary fissure and anterior lobe differs significantly from the flocculonodular lobe [*F*_(2,6)_ = 6.94, *p* = 0.03].

**Figure 8.**
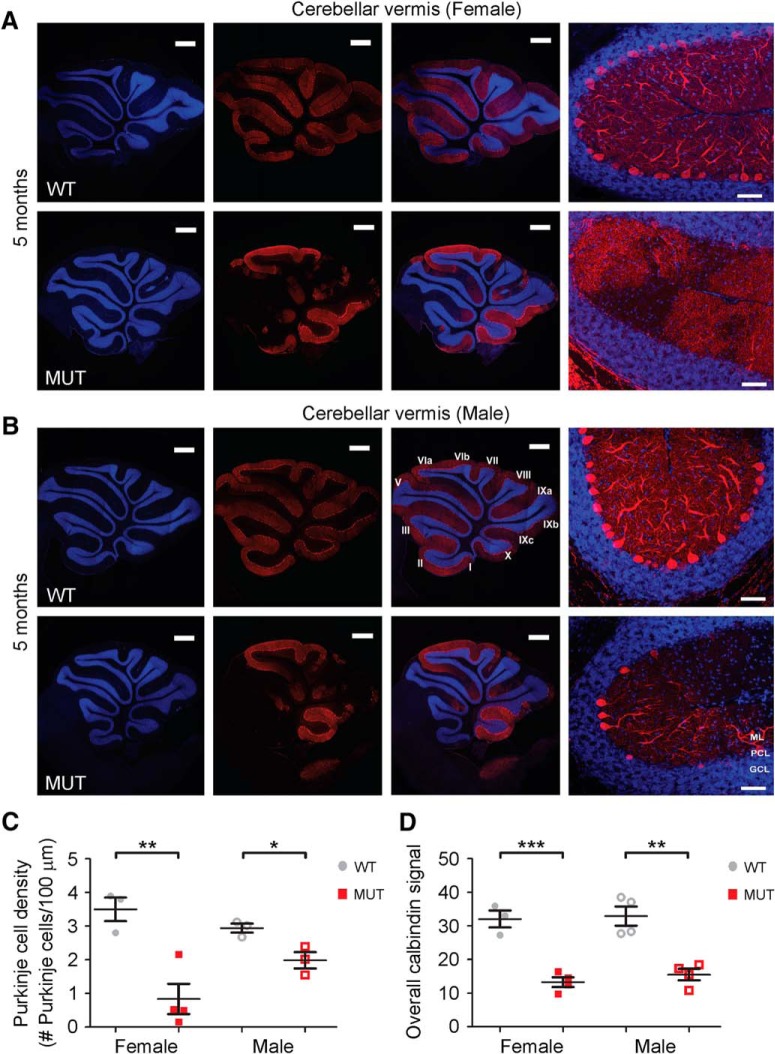
PC loss in *Nhe6*-null mouse cerebellum, female and male. ***A***, ***B***, Representative images of midsagittal sections of whole cerebellar vermis and the vermal primary fissure immunostained with the PC marker calbindin (red) in WT and MUT female (***A***) and male (***B***) mice at 5 mo of age. Lower-magnification images depicting lobules I through X are shown in the left and middle panels, and higher-magnification images depicting the primary fissure are shown in the rightmost panels. Nuclei were stained with DAPI (blue). ML, molecular layer; PCL, Purkinje cell layer; GCL, granule cell layer. Scale bars, 500 μm (left and middle panels) and 50 μm (right panels). ***C***, ***D***, Graphs depicting quantitative analysis of PC density (***C***) and overall calbindin signal (***D***) in the vermal primary fissure in WT and MUT female and male (from Fig. 4*D*, *E*) mice at 5 mo of age. *Nhe6*-null female mice display significantly decreased PC density (**, *p* = 0.007, ***C***) and overall calbindin signal (***, *p* = 0.001, ***D***) compared with WT female mice. As shown in Fig. 4*D*, *E*, *Nhe6*-null male mice at 5 mo of age display significantly decreased PC density (*, *p* = 0.03, ***C***) and overall calbindin signal (**, *p* = 0.002, ***D***), respectively, compared with WT male mice. There are no significant differences between *Nhe6*-null female and male mice. WT *n* = 3, MUT *n* = 4 for female (***C*** and ***D***); WT *n* = 3, MUT *n* = 3 for male (***C***); WT *n* = 4, MUT *n* = 4 for male (***D***). Data are presented as mean ± SEM. Statistical analyses were conducted using two-tailed Student’s *t* tests.

Finally, in the exon 6 *Nhe6*-null model, we compared hemizygous *Nhe6*-null males to homozygous *Nhe6*-null females so as to ascertain the severity of effect in males versus females. We examined homozygous *Nhe6*-null female mice at 5 mo of age, wherein we observe statistically significant differences in PC density (*p* = 0.007) and overall calbindin signal (*p* = 0.001) between wild-type and mutant female mice; however, we do not observe strong or statistically significant differences between hemizygous *Nhe6*-null male mice and homozygous *Nhe6*-null female mice (Fig. 8). In summary, our findings demonstrate that NHE6 loss-of-function mutations consistently result in PC loss, as evidenced using two distinct *Nhe6*-null mouse models based on two independent targeting events and through analysis of male and female mice in one model. Combined, the results suggest a time course of PC loss accelerating after 2 mo and an interesting spatial pattern involving the vermal primary fissure and anterior lobe, but sparing the vermal flocculonodular lobe.

### *Astrocytic and strong microglial reactivity in aging* Nhe6*-null brain pinpoints axonal tracts as site of major pathology*

**Figure 9.**
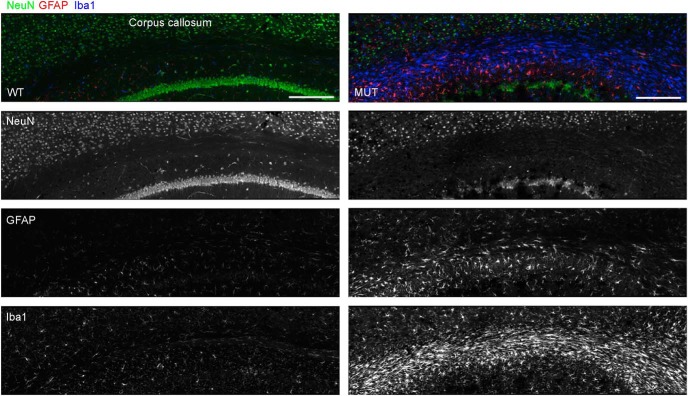
Microglia in the corpus callosum of aged *Nhe6*-null mouse brain. Representative images of 30-μm coronal brain sections through the CC from 22-mo-old WT and MUT male littermate mice after triple immunohistochemical staining with antibodies against NeuN (green), a marker for neurons; GFAP (red), a marker for reactive astrocytes; and Iba1 (blue), a marker for microglia. Shown are merged images and images of single channels for NeuN, GFAP, and Iba1, respectively. The images reveal strong reactivity of microglia and astrocytes in the CC of aged (22-mo-old) MUT male mice compared with WT male littermates. Scale bars, 200 μm.

**Figure 10.**
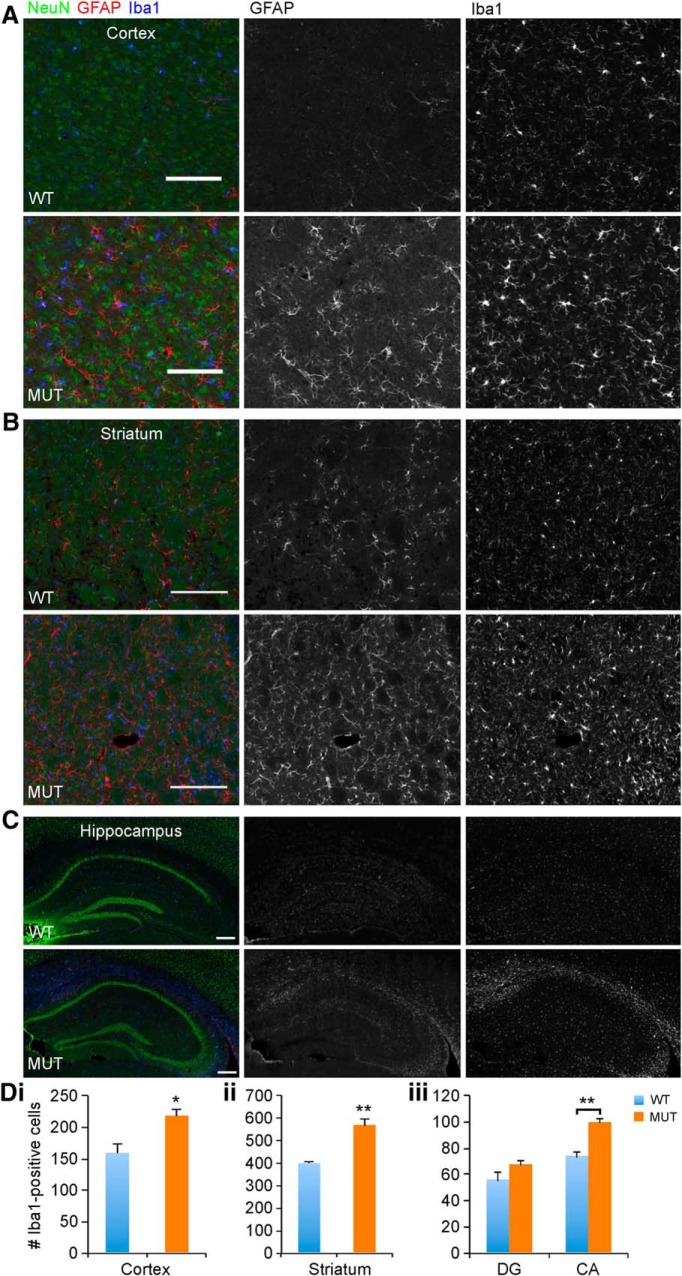
Microglia in the cortex, striatum, and hippocampus of aged *Nhe6*-null mouse brain. ***A***, ***B***, ***C***, Representative images of 30-μm coronal brain sections from 22-mo-old WT and MUT male littermate mice after triple immunohistochemical staining with antibodies against NeuN (green), a marker for neurons; GFAP (red), a marker for astrocytes; and Iba1 (blue), a marker for microglia. Shown are merged images and images of single channels for GFAP and Iba1, respectively, for sections of cortex (***A***), striatum (***B***), and hippocampus (***C***). The images reveal gliosis, as indicated by Iba1 immunostaining, in the respective brain regions of aged (22-mo-old) MUT male mice compared with WT male littermates. Scale bars, 50 μm (***A*** and ***B***) and 200 μm (***C***). ***Di-iii***, Graphs depicting quantification of gliosis, based on the number of Iba1-positive cells in a region of defined size, in the cortex (***Di***), striatum (***Dii***), and hippocampus (***Diii***) of WT and MUT male littermate mice at 22 mo of age. Microglia density is significantly increased in MUT male mice compared with WT male littermates in the cortex (*, *p* = 0.019, ***Di***), striatum (**, *p* = 0.0054, ***Dii***), and CA region of the hippocampus (**, *p* = 0.0048, ***Diii***) but not in the DG region of the hippocampus (*p* = 0.18). CA, *cornu Ammonis*; DG, dentate gyrus. Images similar to those shown in ***A***, ***B***, and ***C*** were used for quantitation, with the number of microglia in 228-μm^2^ regions being counted and summed across the total number of regions analyzed. The number of 228-μm^2^ regions analyzed for each brain region for each mouse brain were as follows: 16 (cortex), 36 (striatum), 4 (hippocampus, DG), and 6 (hippocampus, CA). WT *n* = 3, MUT *n* = 4. Data are presented as mean ± SEM. Statistical analyses were conducted using two-tailed Student’s *t* tests.

**Figure 11.**
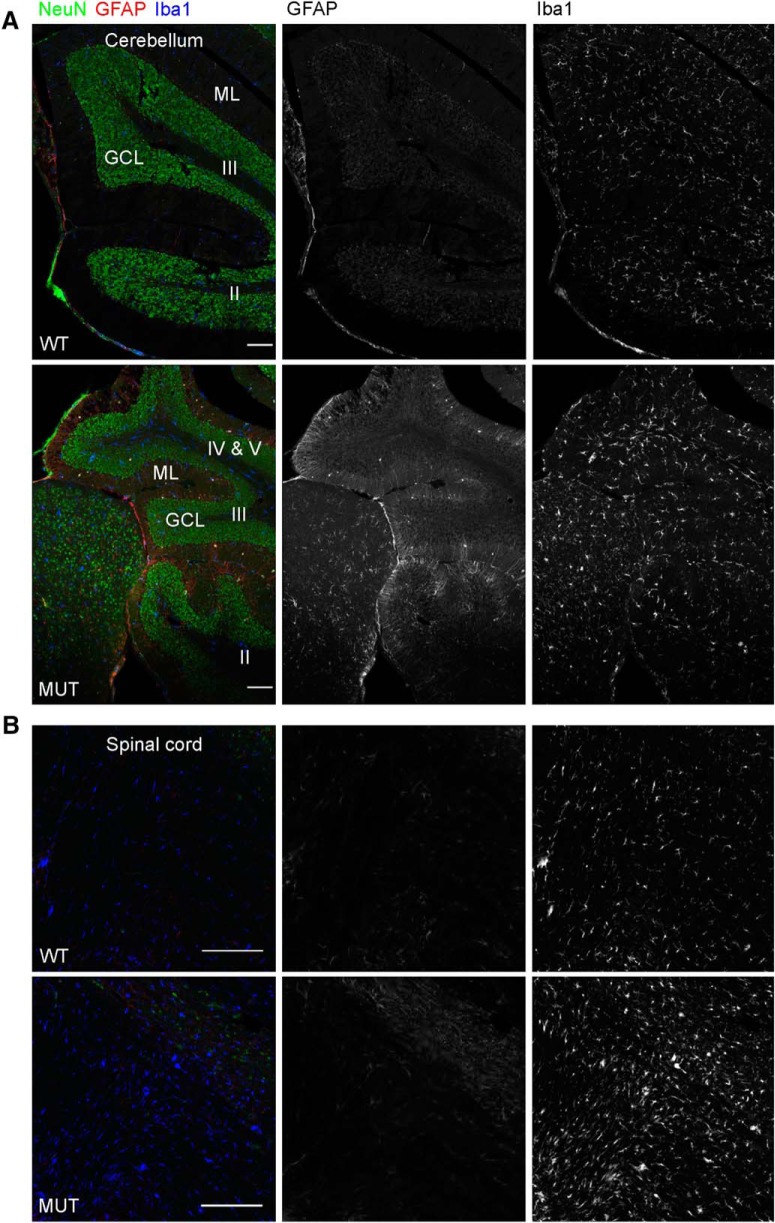
Microglia in the cerebellum and spinal cord of aged *Nhe6*-null mouse. ***A***, ***B***, Representative images of 30-μm sagittal sections from 22-mo-old WT and MUT male littermate mice after triple immunohistochemical staining with antibodies against NeuN (green), a marker for neurons; GFAP (red), a marker for astrocytes; and Iba1 (blue), a marker for microglia. Shown are merged images and images of single channels for GFAP and Iba1, respectively, for sections of cerebellum (***A***) and spinal cord (***B***). The images reveal gliosis, as indicated by Iba1 immunostaining, in the respective regions of aged (22-mo-old) MUT male mice compared with WT male littermates. Roman numerals overlaying images in ***A*** reflect the respective lobules of the vermis folia. GCL, granule cell layer; ML, molecular layer. Scale bars, 100 μm (***A***) and 200 μm (***B***).

Given the possibility for a widespread neurodegenerative mechanism in the brain in CS, we endeavored to identify a component of glial pathology. Neuroinflammatory mechanisms are emerging as important contributors to neurodegenerative pathogenesis, particularly with regard to innate immunity involving microglia (Ransohoff, 2016). To identify and localize pathologic changes in aging *Nhe6*-null mouse brain, we conducted immunohistochemistry with antibodies against GFAP and Iba1, markers of astrocytes and microglia, respectively, using sections of brains from male mice at ∼2 yr (22 mo). In *Nhe6*-null male mouse brain, we observe a prominent astrocytic and microglia response, particularly in major axonal tracts such as the CC (Fig. 9). We also examined additional brain regions, including cortex, striatum, and hippocampus (Fig. 10) and cerebellum and spinal cord (Fig. 11). Quantification of the number of microglia in different brain regions using anti-Iba1 immunohistochemistry demonstrates statistically significant increases in aged *Nhe6*-null male mouse brain compared with wild-type male littermates in all regions analyzed, including in cortex (*p* = 0.019), striatum (*p* = 0.0054), and hippocampus CA region (*p* = 0.0048; Fig. 10). In 22-mo-old *Nhe6*-null cerebellum, we observe a prominent microglial response (Fig. 11*A*, blue) and strong GFAP staining in cells with morphology characteristic of Bergmann glia in the molecular layer (Fig. 11*A*, red). In the spinal cord of mutant animals, we also observe increased Iba1 and GFAP staining in comparison to wild-type littermates (Fig. 11*B*).

**Figure 12.**
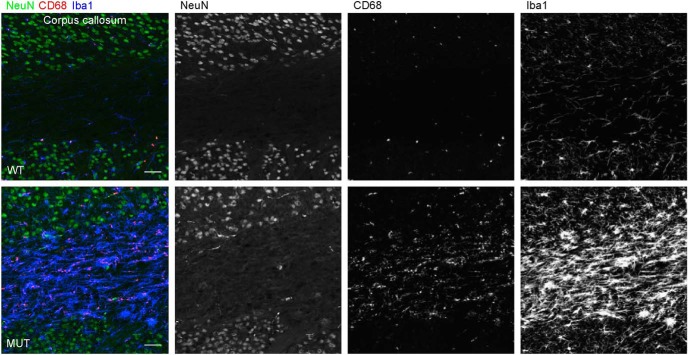
Activated microglia in the corpus callosum of aged *Nhe*6-null mouse brain. Representative images of 30-μm coronal brain sections through the CC of 22-mo-old WT and MUT male littermate mice after triple immunohistochemical staining with antibodies against NeuN (green), a marker for neurons; CD68 (red), a marker for activated microglia; and Iba1 (blue), a marker for microglia. Shown are merged images and images of single channels for NeuN, CD68, and Iba1, respectively. The images reveal a more prominent presence of activated microglia, as indicated by microglia containing large CD68-positive puncta, in the CC of aged (22-mo-old) MUT male mice compared with WT male littermates. Scale bar, 50 μm.

**Figure 13.**
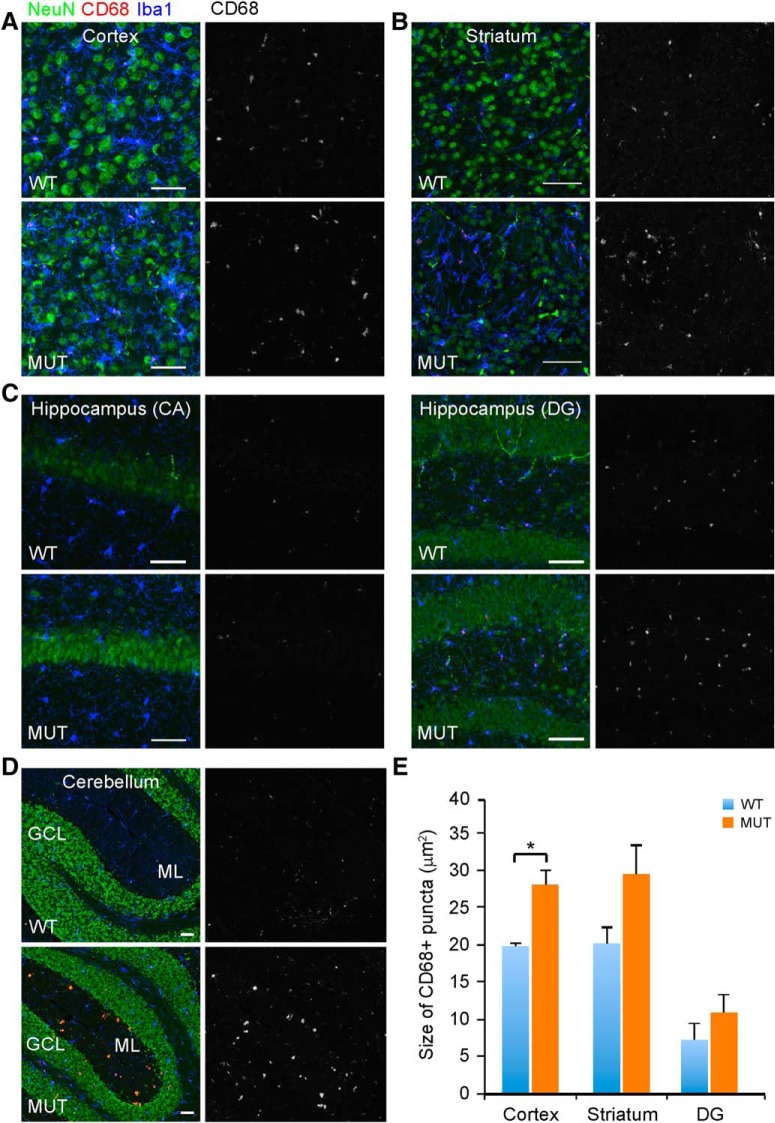
Activated microglia in the cortex, striatum, hippocampus, and cerebellum of aged *Nhe*6-null mouse brain. ***A–D***, Representative images of 30-μm brain sections from 22-mo-old WT and MUT male littermate mice after triple immunohistochemical staining with antibodies against NeuN (green), a marker for neurons; CD68 (red), a marker for activated microglia; and Iba1 (blue), a marker for microglia. Coronal sections were used for cortex (***A***), striatum (***B***), and hippocampus (***C***), and sagittal sections were used for cerebellum (***D***). Shown are merged images and single-channel images for CD68. ML, molecular layer; GCL, granule cell layer. Scale bars, 50 μm. ***E***, Graph depicting quantification of microglial activation, based on the size of CD68-positive puncta in Iba1-positive cells. For hippocampus, the analysis was divided into two subregions, CA and DG. No CD68 size analysis was done in CA, since there were few CD68-positive microglia and the CD68-positive puncta that were present were very small in size. The size of CD68-positive puncta is significantly increased in MUT male mice compared with WT male littermates in cortex (*, *p* = 0.015). Although not reaching statistical significance, an increase in size is also noted in striatum (*p* = 0.11). For the DG of the hippocampus, the size of CD68-positive puncta is not statistically different between MUT male mice and WT male littermates (*p* = 0.34). WT *n* = 3, MUT *n* = 3. Data are mean ± SEM. Statistical analyses were conducted using two-tailed Student’s *t* tests.

We next asked whether the activity state of microglia might be altered in aging brains of *Nhe6*-null animals. Here, we used immunohistochemical staining for CD68, a lysosomal marker reflecting actively phagocytic microglia (Safaiyan et al., 2016), as a means for detecting differences in microglia activity. In 22-mo-old *Nhe6*-null male mouse CC, we observe a substantial increase in CD68 staining within microglia, compared with images from wild-type male littermates (Fig. 12, red). This observation is consistent with a dramatic activation of microglia in aged *Nhe6*-null male mouse brain. Similarly, as reflected by the presence and size of CD68-positive puncta, phagocytic activity is increased in microglia of aged mutant mice in comparison to wild-type littermates in cortex, striatum, hippocampus, and cerebellum (Fig. 13). Interestingly, we observe the presence of microglia with dense and large CD68 staining sparsely distributed in both molecular layers and granular layers of cerebellum (Fig. 13*D*), which may be related to PC loss in the cerebellum.

**Figure 14.**
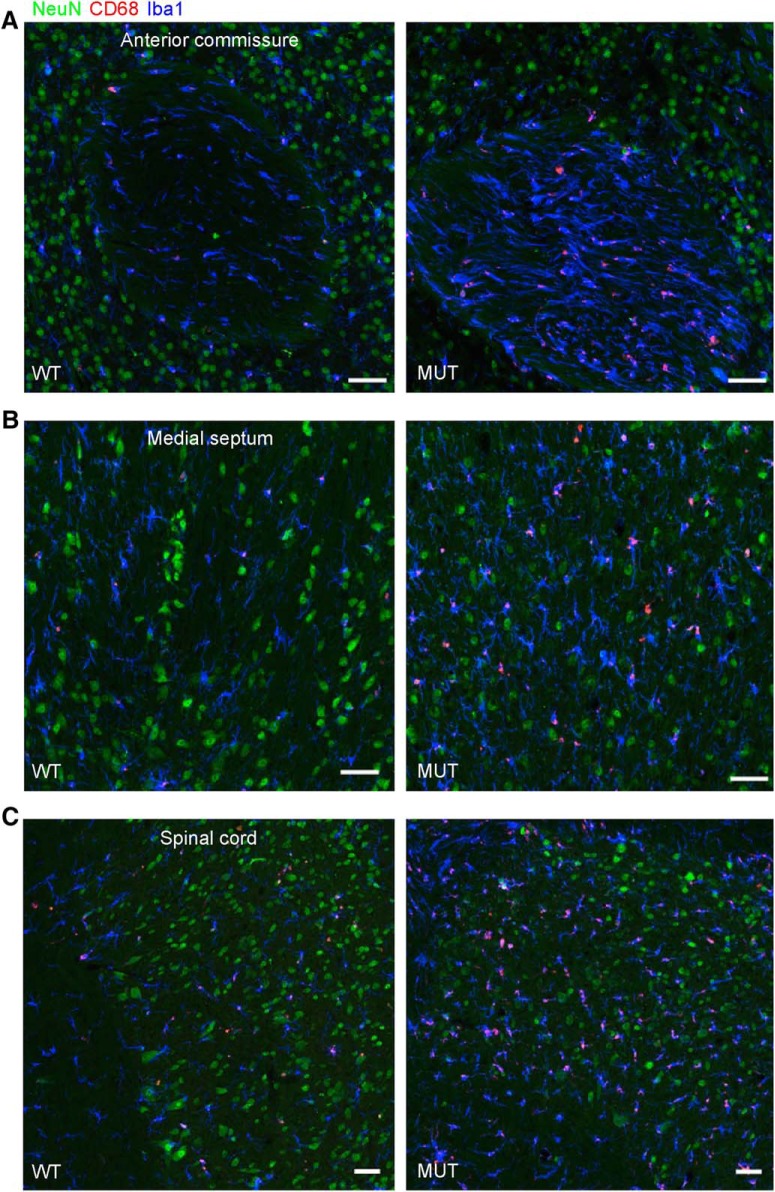
Gliosis in other axonal tracts in aged *Nhe6*-null mouse. ***A–C***, Representative images of 30-μm brain sections from 22-mo-old WT and MUT male littermate mice after triple immunohistochemical staining with antibodies against NeuN (green), a marker for neurons; CD68 (red), a marker for activated microglia; and Iba1 (blue), a marker for microglia. Shown are merged images for coronal sections of the anterior commissure (***A***) and medial septum (***B***), and sagittal sections of the spinal cord (***C***). The images reveal the presence of abnormal gliosis, as indicated by the strong immunostaining for CD68 and Iba1, in axonal tracts of various regions of aged (22-mo-old) MUT male mice compared with WT male littermates. Scale bars, 50 μm.

Given the prominent microglial response in the CC of aged *Nhe6*-null male mice, we investigated the presence and activity of microglia in other major axonal tracts. Based on immunohistochemical staining for Iba1 and CD68, we also observe strong and abnormal staining of microglia in large axonal tracts of other brain regions—namely, the anterior commissure, medial septum, and spinal cord—in aged *Nhe6*-null male mice (Fig. 14). These data are consistent with an innate immune response and neurodegenerative mechanisms in major axonal tracts in *Nhe6*-null brains.

**Figure 15.**
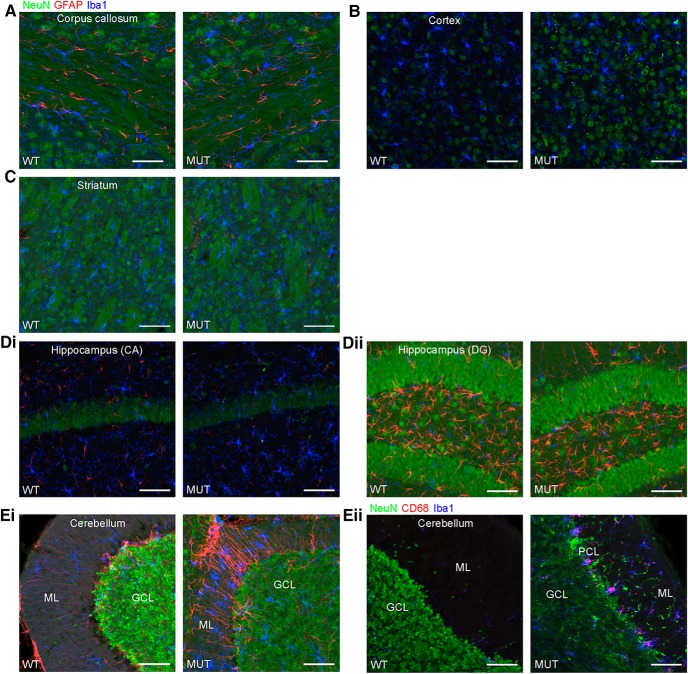
Glial activation in the cerebellum of *Nhe*6-null mice at 2 mo of age. ***A–Eii***, Representative images of 30-μm brain sections from 2-mo-old WT and MUT male littermate mice after triple immunohistochemical staining with antibodies against NeuN (green), a marker for neurons; GFAP (red), a marker for astrocytes (***A–Ei***), or CD68 (red), a marker for activated microglia (***Eii***); and Iba1 (blue), a marker for microglia. Coronal sections were used for CC (***A***), cortex (***B***), striatum (***C***), CA region of the hippocampus (***Di***), and DG region of the hippocampus (***Dii***), and sagittal sections were used for cerebellum (***Ei***and ***Eii***). The amount of gliosis appears similar between MUT male mice and WT male littermates in CC, cortex, striatum, and hippocampus at 2 mo of age (***A–Dii***). However, gliosis in the cerebellum, especially in the ML, is apparent at this age in MUT male mice. This is reflected in the strong signals for GFAP and Iba1 (***Ei***) and CD68 and Iba1 (***Eii***), the latter of which is indicative of activated microglia. ML, molecular layer; PCL, Purkinje cell layer; GCL, granule cell layer. Scale bars, 50 μm.

Using immunohistochemical staining for glial pathology, we pinpointed the emergence of this component of brain pathology in male *Nhe6*-null mouse brains in greater detail. Consistent with the phenomenon of lack of statistically significant differences between wild-type and mutant animals in thickness or areas of different brain regions at 2 mo (outside of the cerebellum) based on histologic measures (Fig. 3), we do not observe abnormal increases in astrocytic staining (GFAP) or microglial staining (Iba1) in the CC, cortex, striatum, or hippocampus at this time point (Fig. 15*A–Dii*). However, we observe prominent glial responses, including activated Bergmann glia, in cerebellum in 2-mo-old *Nhe6*-null male mice (Fig. 15*Ei*). Furthermore, microglia are phagocytically active, as indicated by the presence of CD68-positive puncta (Fig. 15*Eii*). These data are most consistent with an earliest onset of degenerative changes in the cerebellum at ∼2 mo postnatal in *Nhe6*-null mouse. Notably, in later stages of postnatal life, the most prominent microglial responses appear in major axonal tracts. An overall summary of the trajectories of brain pathology in *Nhe6*-null male mouse across the postnatal lifespan is presented in Table 3.

**Table 3.** Summary of phenotypic findings

Region and analysis	Birth	1 mo	2 or 5 mo	1 or 2 yr
Whole brain				
Gross	No change (Fig. 1)	No change (Fig. 1)	At 2 mo, reduced size relative to control (Fig. 1)	At 2 yr, reduced size relative to control and 2-mo time point (Fig. 1)
Cerebellum				
Gross	No change (Fig. 1)	No change (Fig. 1)	At 2 mo, reduced size relative to control (Fig. 1)	At 2 yr, reduced size relative to control and 2-mo time point (Fig. 1)
Tissue area (histology)	NA	NA	At 2 mo, reduced relative to control (Fig. 3)	At 2 yr, reduced relative to control and 2-mo time point (Figs. 2 and 3)
PC loss (IHC)	NA	NA	At 2 mo, some potential loss of PC but highly variable (Fig. 4); at 5 mo, approximate 10% loss (Fig. 4)	At 1 yr, approximate 80% loss (Fig. 4)
Astrocytes/microglia (IHC)	NA	NA	At 2 mo, increased (Fig. 15)	At 2 yr, increased (Fig. 11)
Activated microglia (IHC)	NA	NA	At 2 mo, increased (Fig. 15)	At 2 yr, increased (Fig. 13)
Cortex/cerebrum				
Gross	No change (Fig. 1)	No change (Fig. 1)	At 2 mo, reduced size relative to control (Fig. 1)	At 2 yr, reduced size relative to control and 2-mo time point (Fig. 1)
Cortical thickness (histology)	NA	NA	At 2 mo, similar thickness to control (Fig. 3)	At 2 yr, reduced relative to control but not significantly reduced relative to 2-mo time point (Figs. 2 and 3)
Astrocytes/microglia (IHC)	NA	NA	At 2 mo, not increased (Fig. 15)	At 2 yr, increased (Fig. 10)
Activated microglia (IHC)	NA	NA	NA	At 2 yr, increased (Fig. 13)
Hippocampus				
Tissue area (histology)	NA	NA	At 2 mo, similar area to control (Fig. 3)	At 2 yr, reduced relative to control but not reduced relative to 2-mo time point (Figs. 2 and 3)
Astrocytes/microglia (IHC)	NA	NA	At 2 mo, not increased (Fig. 15)	At 2 yr, increased in CA region (Fig. 10)
Activated microglia (IHC)	NA	NA	NA	At 2 yr, increased in DG region (not statistically significant; Fig. 13)
Striatum				
Tissue area (histology)	NA	NA	At 2 mo, similar area to control (Fig. 3)	At 2 yr, reduced relative to control but not reduced relative to 2-mo time point (Figs. 2 and 3)
Astrocytes/microglia (IHC)	NA	NA	At 2 mo, not increased (Fig. 15)	At 2 yr, increased (Fig. 10)
Activated microglia (IHC)	NA	NA	NA	At 2 yr, increased (not statistically significant; Fig. 13)
Spinal cord				
Tissue area (histology)	NA	NA	NA	At 2 yr, reduced relative to control (Fig. 2)
Astrocytes/microglia (IHC)	NA	NA	NA	At 2 yr, increased (Figs. 11 and 14)
Activated microglia (IHC)	NA	NA	NA	At 2 yr, increased (Fig. 14)
Major axonal tracts				
Corpus callosum				
Astrocytes/microglia (IHC) Activated microglia (IHC)	Strong NHE6 protein expression (Ouyang et al., 2013)	NA	At 2 mo, no increase in astrocytes/microglia (Fig. 15)	At 2 yr, increased astrocytosis, microgliosis, and activated microglia (Figs. 9 and 12)
Anterior commissure				
Microglia (IHC) Activated microglia (IHC)	Strong NHE6 protein expression (Ouyang et al., 2013)	NA	NA	At 2 yr, increased microgliosis and activated microglia (Fig. 14)
Medial septum				
Microglia (IHC) Activated microglia (IHC)	Strong NHE6 protein expression (Ouyang et al., 2013)	NA	NA	At 2 yr, increased microgliosis and activated microglia (Fig. 14)

CA, *cornu Ammonis*; DG, dentate gyrus; IHC, immunohistochemistry; mo, month or months; NA, not applicable; PC, Purkinje cell; yr, year or years.

## Discussion

Mutations in endosomal NHE6 (also known as SLC9A6) are associated with intellectual disability, postnatal microcephaly, absent speech, ataxia with progressive cerebellar atrophy, epilepsy, and neurologic regression (Christianson et al., 1999; Pescosolido et al., 2014). A prior study showed that *Nhe6*-null mice exhibit developmental defects such as reduced levels of neuronal arborization, functional connectivity, and mature synapses (Ouyang et al., 2013). Prior studies have also demonstrated loss of PCs and neuropathological findings consistent with endolysosomal pathology (Strømme et al., 2011). Overall, our data here, combined with prior studies, indicate that the *Nhe6*-null mouse model has very strong validity for the human disorder. In the present study, we demonstrate important trajectories of change in brain measurements. These trajectories strongly suggest mixed neurodevelopmental and neurodegenerative pathology, as revealed by both prominent brain undergrowth as well as progressive brain volume loss most profound in the cerebellum in *Nhe6*-null mice. The cerebellar findings are strongly in line with the human condition (Christianson et al., 1999; Garbern et al., 2010; Pescosolido et al., 2014). We also observe a strong astrocytic and microglial response throughout the aging male mouse brain that is particularly impressive in major axonal tracts. The results suggest a neurodegenerative process in aging *Nhe6*-null mouse brain extending beyond the cerebellum alone and, in particular, provide strong support for a primary axonal pathology and only more mild generalized progressive atrophy. These data in this excellent mouse model of the clinical condition are valuable given the constraints on discerning progressive neuropathology in rare genetic disorders where there are presently limitations in available patient neuroimaging and postmortem material.

The interpretation of brain undergrowth in the mouse mutant is also highly consistent with the clinical observations of postnatal microcephaly and developmental trajectories in early childhood in boys with CS. Postnatal microcephaly is reduction in growth of head circumference in the early postnatal period and is generally construed to reflect undergrowth of brain tissue as a result of decreased neuronal arborization, frequently reflecting organellar biology or gliogenesis (Passemard et al., 2017; van Dyck and Morrow, 2017). In some situations, postnatal microcephaly may reflect early aggressive degenerative pathology. In this later situation, pure degenerative pathology is frequently associated with initially normal cognitive, motor, and adaptive development followed by a prominent loss of skills and strong inflection in slope of head circumference growth with increase in cerebrospinal fluid space (Gilmore and Walsh, 2013). In patients with CS, most common developmental histories involve early global developmental delays. Growth trajectories in head circumference are reduced, but the slope of increase generally follows a slope similar to that of typically developing children without a strong downward inflection in slope that would suggest early cerebral neurodegeneration (Pescosolido et al., 2014). Again, the clinical syndrome is often overlapping with AS, another postnatal microcephaly syndrome without a known neurodegenerative component (Dagli et al., 1998 [updated 2015]). That said, there are certainly regressions in CS; however, whether regressions reflect neurodegenerative pathology is unclear. There are also examples of patients with MRI data consistent with some atrophy outside the cerebellum such as in the midbrain or other brain regions (Gilfillan et al., 2008; Pescosolido et al., 2014). There is one report consistent with a retinal degenerative condition (Mignot et al., 2013).

Cerebellar atrophy was among the first documented features in the original CS pedigree report and is among the most common neuroimaging findings in CS cases (Christianson et al., 1999; Bosemani et al., 2014). Approximately 85% of CS patients suffer from truncal ataxia, which is often associated with cerebellar dysfunction. This progressive cerebellar atrophy is a distinct feature of CS that may be used to distinguish CS from AS (Pescosolido et al., 2014). The risk for developing cerebellar atrophy appears to increase after the first decade of life, suggesting that there is progressive cerebellar degeneration in patients with CS (Pescosolido et al., 2014). Indeed, in the CS mouse model, the cerebellum is the earliest region to demonstrate neurodegenerative pathology, and the cerebellar atrophy and PC loss appear to progress with aging. Structural MRIs in CS patients have identified cerebellar abnormalities such as moderate to severe cerebellar atrophy in both the hemispheres and vermis, as well as bilateral lesions in the inferior cerebellum with minimal area loss (Pescosolido et al., 2014). Earlier studies have revealed that *Nhe6*-null mouse models (e.g., hemizygous male and homozygous females) exhibit PC degeneration as early as P57 (Strømme et al., 2011; Sikora et al., 2016). In the current study, we replicate and extend prior studies by characterizing the temporal and spatial distribution of PC loss in two *Nhe6*-null mouse models. We demonstrate severe PC loss in the anterior lobe and primary fissure of the vermis, but not in the flocculonodular lobe of the vermis, and this cell loss progresses with age. We also observe similar patterns of PC loss using a new *Nhe6*-null mouse model with a different gene mutation. Overall, data from human and mouse together appear to reflect mixed developmental and degenerative pathology. At the clinical level, the degenerative pathology seems somewhat variable and generally slowly progressive, as described initially by Christianson and more recently by others as well (Christianson et al., 1999; Pescosolido et al., 2014).

In the mouse model, our results reveal prominent gliosis, notable in the CC (corpus callosum), which suggests that NHE6 mutation may be associated with primary axonal pathology. Consistent with this, we also observe prominent gliosis in various additional large axonal tracts, namely, in the anterior commissure, medial septum, and spinal cord. The distribution of this pathology is highly consistent with strong protein expression of NHE6 in developing axonal tracts (Ouyang et al., 2013). Although few in number, several CS patients have shown thinning of the CC on MRI study (Garbern et al., 2010). This result is also consistent with other endosome/vesicle disorders wherein axonal pathology is seen (Tsang et al., 2009; Harbour et al., 2010; Allison et al., 2013).

Our study also pinpoints a prominent glial response, by both astrocytes and microglia. This result is important, as these patterns of glial activity are consistent with a neurodegenerative process outside the cerebellum that appears to occur later and is generally widespread. Here, we observed a prominent molecular change reflected by the upregulation of Iba1 and CD68. A microglial response can serve as a marker for neurodegeneration and may also be a target for therapeutic intervention (Ransohoff, 2016). The timing of the microglia response again appears also to support a model of initial undergrowth followed later by neurodegeneration in the mutant, as there is widespread decrease in volumes evident by 2 mo yet the gliosis is not evident at this time point outside of the cerebellum.

We have also used mathematical modeling to consider the contributions of undergrowth, neurodegeneration, or both mechanisms to explain our data. We believe that the mathematical modeling most strongly supports mixed mechanisms. In both the cerebrum and cerebellum, the degeneration-only model will fit the data only in the situation that there are two strong phases of degeneration, which seems implausible. The first phase of degeneration would need to occur very early, at 1–2 mo, and this is not supported by the histologic data; for example, the gliosis is not prominent until after 2 mo of age. The modeling data would need to invoke a rapid early degeneration, then a long stall in neurodegeneration (even with resurgent growth in cerebrum), followed by another phase of neurodegeneration. Finally, while not a focus of this study, we do not see an obvious increase in lateral ventricle size as seen in other mouse models with prominent cerebral neurodegeneration. In the cerebellum, PC loss is also not prominent at this very early stage; even undergrowth of the cerebellum seems likely, which is consistent with the clinical observations of an early ataxia often seen in the absence of any MRI finding in cerebellum. Therefore, the bulk of the data, from both mouse and clinical studies, seem to support an initial phase of developmental delays subsequently superseded by a degenerative phase. Indeed, the cerebellum data fit this sort of model. However, our mathematical modeling inevitably oversimplifies the complex biology, as these two phases of delayed development and enhanced degeneration are likely overlapping to some extent, particularly in the adolescent period. Further, while the cerebrum data fit the undergrowth-only model quite well, there is likely both undergrowth and degeneration even in the region of the cerebrum, and this interpretation is supported by the observations of increased reactive microglia in regions of the brain beyond the cerebellum alone such as in the cortex.

Our study has various strengths and a few limitations. With regard to strengths, our study has investigated trajectories of brain changes across the postnatal lifespan and therein provides support for both an undergrowth of brain regions and neurodegenerative processes. In various regions of brain, particularly in cortex, hippocampus, and striatum, the data are most consistent with the largest component due to reduction of growth, i.e., plateau in tissue volume changes, as opposed to a strong downward slope reflecting loss. The cerebellum appears to show the strongest and earliest decline in tissue area, reflecting a clear neurodegenerative process. The findings of a strong glial response provide substantial support for a neurodegenerative process in CS. Another strength of our study is the investigation of the CS mouse model into advanced age, i.e., ∼2 yr of age. Our results stand in complement to the few existing postmortem studies in human patients with CS. The two existing postmortem studies both demonstrate loss of PCs, as well as moderate atrophy of the cerebellum (Christianson et al., 1999; Garbern et al., 2010). Additionally, the study by Garbern et al. demonstrates generalized atrophy of caudate, putamen, and globus pallidus, with relatively less prominent changes in cortex. This study also demonstrated widespread neuronal and glial tau pathology, which is not easily observable in mouse models given a general lack of conservation of these protein aggregates in murine models. A question has been raised as to whether the in-frame deletion in this family is distinct from the typical loss-of-function mutations found in CS (Ilie et al., 2014), raising the question as to whether these neuropathological findings are generalizable. Certain aspects of our study are consistent with this prior postmortem study, including, in particular, widespread glial responses as well as pathology in major fiber tracts such as the CC. Our study has the limitation that the trajectories of changes in brain size are based on interpretations from cross-sectional measurements in brains from different animals at isolated time points. Moving forward, an additional study will be to investigate brain size changes longitudinally using MRI small animal imaging in the same living animal across the postnatal lifespan.

In summary, our study pinpoints dynamic changes in brain areas during postnatal life in *Nhe6*-null mouse, likely reflecting a combination of brain undergrowth and neurodegenerative processes. These processes are prominent and early in the cerebellum, but also widespread across the brain. Moreover, a strong microglial response, particularly in large axonal tracts, is involved, which is consistent with a primary axonal pathology. In this way, the mouse model of CS provides important insight into the progressive aspects of this rare genetic condition with aging, and these pathologies may provide substantial insight into more common developmental and neurodegenerative conditions.

## Synthesis

Reviewing Editor: Lorna Role, Stony Brook Decisions are customarily a result of the Reviewing Editor and the peer reviewers coming together and discussing their recommendations until a consensus is reached. When revisions are invited, a fact-based synthesis statement explaining their decision and outlining what is needed to prepare a revision will be listed below.

This study delineates postnatal neurodevelopmental changes (from 1 month to 2 years) in the gross morphology and histopathology of the brains of mice containing a null mutation of the endosomal Na+/H+ exchanger 6 gene (NHE6/SLC9A6), an established animal model of Christianson Syndrome (generated by transgene lacZ/Neo insertion into exon 6).

The authors demonstrate that the NHE6¬-null mouse exhibit measurably diminished neuronal growth, as well as gross tissue loss, in several brain regions (i.e., cortex, striatum, hippocampus and cerebellum). The most prominent changes were observed in the cerebellum - which exhibited both reductions in growth and marked neurodegeneration.

An additional new finding was the increase in density of activated astrocytes and microglia in several regions and especially in large axon tracts including the corpus callosum, anterior commissure, medial septum, and spinal cord, indicative of a pronounced neuroinflammatory response.

The investigators have also engineered another NHE6¬-null mouse model where the lacZ/Neo transgene replaced exons 2 and 3 of NHE6 and, obtained comparable results albeit in a more limited analysis.

While many of the data presented are largely a corroboration of earlier published studies (Stromme et al, 2011; Ouyang et al., 2013; Sikora et al. 2016), an original and significant finding is the observation of a pronounced neuroinflammatory response in the NHE6-null mouse which had not been previously documented. In addition, this paper makes a significant effort to go beyond a description of qualitative changes by immunohistochemistry to quantify some of those changes.

Comments and queries:

-Could the authors clarify the rationale for generating another NHE6¬-null mouse model (cross-breed to the same C57Bl/6J background as the original NHE6¬-null mouse) given that the only difference is the site of insertion of the transgene. Please discuss why one might anticipate a different outcome from the original NHE6¬-null mouse strain.

Minor Comments:

1. Line 233; the authors' state that with respect to the cerebrum, “... a strong reduction in the rate of increase exists between 1 to 2 months”. This conclusion is largely based on one data point and therefore seems overstated, especially since this difference is largely overcome by 6 months. A tempered adjective would be more fitting.

2. In Figs 12 and 13, the red staining is more difficult to see. Perhaps use grayscale for easier visualization.

## Author Response

Manuscript Instructions

Instruction: The sex of the species studied is not mentioned in the materials and methods section. Please mention “male”, “female”, “x males and x females”, “of either sex”.

Response: We have now added the following sentences under the “Animal procedures” subsection of “Materials and Methods”:

“In general, matched male wild-type and mutant littermate pairs were used for experiments, as CS is an X-linked condition affecting males. Matched female wild-type and mutant littermate pairs were used for one set of studies (Fig. 8). Numbers of animals used for the various studies are stated in Table 1 and the figure legends.” (Page 5, Lines 97-100)

Instruction: eNeuro is pleased to be part of the Resource Identification Initiative, a project aimed at clearly identifying the key resources used in the course of scientific research. ...We see these as important steps toward ensuring reproducible methods and providing critical data to help researchers identify suitable reagents and tools, and we are now asking authors to include RRIDs in their manuscripts.

Response: In furthering eNeuro's participation in the Resource Identification Initiative, we have now inserted RRIDs for the previously published Slc9a6 mouse model and commercially available antibodies. (Pages 5-8, Lines 102, 126-127, and 161-166)

Comments and Queries

Query: Could the authors clarify the rationale for generating another NHE6Â¬-null mouse model (cross-breed to the same C57Bl/6J background as the original NHE6Â¬-null mouse) given that the only difference is the site of insertion of the transgene. Please discuss why one might anticipate a different outcome from the original NHE6Â¬-null mouse strain.

Response: Thank you for the opportunity to clarify this. The mutation that is most widely studied is in exon 6. This mutation is thought to be a null mutation, based on early truncation and/or nonsense-mediated decay of mRNA. Diminished mRNA is shown nicely in Stromme et al. (2011) and loss of protein is shown in Ouyang et al. (2013). An allelic series (i.e., second additional mutation) is always a prudent experiment so as to garner additional proof that mutations are indeed loss-of-function. For example, theoretically, the possibility exists for the Slc9a6 exon 6 mutation that an N-terminal protein could be made that could have dominant-negative activity. (At present, antibodies available for detection of NHE6 are designed against C-terminal peptides and thus likely would not recognize a truncated protein.) The early exons 2/3 mutation is very unlikely to lead to a functional truncated protein. Our data indicating identical phenotypes in the context of two distinct Slc9a6 mutations, particularly in the cerebellum, including the pattern of loss of Purkinje cells, is strong proof that both mutations lead to complete loss-of-function. I believe that people in the field working heavily with either of these models will find these data reassuring.

Comment 1: Line 233; the authors' state that with respect to the cerebrum, “... a strong reduction in the rate of increase exists between 1 to 2 months”. This conclusion is largely based on one data point and therefore seems overstated, especially since this difference is largely overcome by 6 months. A tempered adjective would be more fitting.

Response: Thank you for this comment. We have now removed the adjective “strong” so as to temper the statement. The sentence now reads:

“Although there is continued growth of the cerebrum in Nhe6-null animals, a reduction in the rate of increase exists between 1 to 2 months as well as reduced size in mutants overall.” (Page 10, Lines 239-240)

Comment 2: In Figs 12 and 13, the red staining is more difficult to see. Perhaps use grayscale for easier visualization.

Response: Thank you for this comment as well. For consistency, as well as ease in visualizing and discerning staining, we have converted all single-channel images for Figs. 9-13 to grayscale. Also, we have added in additional figure labels to indicate the stains reflected in the different grayscale images.
